# Comparative Transcriptome Analysis of Adipose Tissues Reveals that ECM-Receptor Interaction Is Involved in the Depot-Specific Adipogenesis in Cattle

**DOI:** 10.1371/journal.pone.0066267

**Published:** 2013-06-21

**Authors:** Hyun-Jeong Lee, Mi Jang, Hyeongmin Kim, Woori Kwak, WonCheoul Park, Jae Yeon Hwang, Chang-Kyu Lee, Gul Won Jang, Mi Na Park, Hyeong-Cheol Kim, Jin Young Jeong, Kang Seok Seo, Heebal Kim, Seoae Cho, Bo-Young Lee

**Affiliations:** 1 Division of Animal Genomics and Bioinformatics, National Institute of Animal science, Rural Development Administration, Suwon, Republic of Korea; 2 Department of Agricultural Biotechnology, Animal Biotechnology Major, and Research Institute for Agriculture and Life Science, Seoul National University, Seoul, Republic of Korea; 3 Interdisciplinary Program in Bioinformatics, Seoul National University, Seoul, Republic of Korea; 4 C&K genomics, Seoul National University Research Park, Seoul, Republic of Korea; 5 Department of Animal Science and Technology, College of Life Science and Natural Resources, Sunchon National University, Jeollanam-do, Republic of Korea; The Scripps Research Institute, United States of America

## Abstract

Adipocytes mainly function as energy storage and endocrine cells. Adipose tissues showed the biological and genetic difference based on their depots. The difference of adipocytes between depots might be influenced by the inherent genetic programing for adipogenesis. We used RNA-seq technique to investigate the transcriptomes in 3 adipose tissues of omental (O), subcutaneous (S) and intramuscular (I) fats in cattle. Sequence reads were obtained from Illumina HiSeq2000 and mapped to the bovine genome using Tophat2. Differentially expressed genes (DEG) between adipose tissues were detected by EdgeR. We identified 5797, 2156, and 5455 DEGs in the comparison between OI, OS, and IS respectively and also found 5657 DEGs in the comparison between the intramuscular and the combined omental and subcutaneous fats (C) (FDR<0.01). Depot specifically up- and down- regulated DEGs were 853 in S, 48 in I, and 979 in O. The numbers of DEGs and functional annotation studies suggested that I had the different genetic profile compared to other two adipose tissues. In I, DEGs involved in the developmental process (eg. *EGR2*, *FAS*, and *KLF7*) were up-regulated and those in the immune system process were down-regulated. Many DEGs from the adipose tissues were enriched in the various GO terms of developmental process and KEGG pathway analysis showed that the ECM-receptor interaction was one of commonly enriched pathways in all of the 3 adipose tissues and also functioned as a sub-pathway of other enriched pathways. However, genes involved in the ECM-receptor interaction were differentially regulated depending on the depots. Collagens, main ECM constituents, were significantly up-regulated in S and integrins, transmembrane receptors, were up-regulated in I. Different laminins were up-regulated in the different depots. This comparative transcriptome analysis of three adipose tissues suggested that the interactions between ECM components and transmembrane receptors of fat cells depend on the depot specific adipogenesis.

## Introduction

Fat is a loose connective tissue composed of adipocytes and functions as not only the main site of energy storage but also the highly active metabolic and endocrine organ. It is restricted to certain areas in the body such as subcutaneous layer between muscle and dermis, around internal organs and between muscles. The amount and distribution of fat are important factors that influence the meat quality in the beef industry [1], [2]. For several decades, it has been known that adipose tissues are regionally heterogeneous with respect to metabolic activities and functions [3]–[5]. Evidences showed that the numerous biological and genetic differences among adipose tissues depend on their anatomical locations [6]–[8]. Adipocytes isolated from different depots differ in size, lipoprotein lipase release, lipid synthetic capacity, fatty acid incorporation and other characteristics [9]–[13]. Regional variations have been also observed in the replicative potential, fatty acid transfer and adipogenic differentiation of preadipocytes originating from various depots of the same individuals under the identical *in vitro* conditions [14]–[18]. The inter-depot variations might have the influences of the inherent genetic programming of the adipocyte development and the microenvironment of adipose depots such as their hormonal response, local nutrient availability, innervations and anatomic constraints [14]–[18]. The fat deposition in muscle, intramuscular fat, might be regulated by the genetic mechanisms different from that in other adipose depots for the occurrence and development. Intramuscular fat content in meat is one of important traits that influence eating quality such as meat tenderness, juiciness, and taste [19]. However, very little is known on the genetic mechanism of intramuscular fat because it is the most difficult depot to study developmentally. Especially, there is the lack of information concerning the transcriptomic differences among omental, subcutaneous and intramuscular adipose tissues.

Gene expression profiling or transcriptome analysis provides new insights to understand the molecular basis of adipogenesis [20]. A few of transcriptome studies in adipose tissues has been reported in cattle using microarray and 3′ digital gene expression-tag analysis [21], [22] The application of next-generation sequencing (NGS) to this area have started to reveal the complexities of different biological processes at an unprecedented level of sensitivity and accuracy [20]. In the present study, we investigated the difference of the depot specific gene expression from 3 different adipose tissues of omental, subcutaneous and intramuscular tissues in cattle using RNA-seq technology.

## Methods

### Ethics Statement

All animal care and experimental procedures were reviewed and approved by the Institutional Animal Care and Use Committee of the National Institute of Animal Science (No. 2010-042).

### Animals

Nine heads of Hanwoo (Korean native cattle, *Bos taurus coreanae*) cattle were fed and managed in the feeding barn at Hanwoo Experimental Station in National Institute of Animal Science under the high quality beef production program (2007). The steers used for this study were 30 months old at slaughter (Body Weight = 835±15.2 kg).

### Sample Preparation

Immediately, after stunning and exsanguination, the muscle and fat portions between the 6th to 7th ribs were removed, and the subcutaneous and intramuscular fat depots were sampled from this rib section. The omental adipose tissue was taken within the lesser curvature of the abomasums and stored at −80°C freezer until the analysis.

### RNA-seq Library Preparation and Sequencing Analysis

Total RNAs of subcutaneous, intramuscular, and omental fat tissues were isolated using TRIzol (Invitrogen) and a RNeasy RNA purification kit with DNase treatment (Qiagen). mRNA was isolated from the total RNA using oligo-dT beads and were reverse transcribed into double strand cDNA fragments. Constructing and sequencing RNA-seq library for each sample were carried out based on protocols of Illumina HiSeq2000 to generate 90 pair-end reads. Quality of RNA-seq reads from all of the adipose tissues was checked using FastQC (Figure S1). The reads passed the quality control were mapped to Bovine Taurus genome (bosTau6) from UCSC using Tophat2 (v2.0.2) and were counted using HTseq (v0.5.3p3). The RNA sequencing data from this study have been submitted to the NCBI Gene Expression Omnibus (GEO) under the accession number of GSE39618 (http://www.ncbi.nlm.nih.gov/geo/).

### Statistical Analysis and DEG Identification

EdgeR was used to identify differentially expressed genes between adipose tissues [23]. This package is designed for the analysis of replicated count-based expression data and is based on a negative binomial model. RNAseq data may exhibit more variability than expected in a poisson distribution because it is more widely dispersed in the genome. When a negative binomial model is used, the dispersion has to be estimated before the DEG analysis is carried out. EdgeR provided Cox-Reid profile-adjusted likelihood method to estimate dispersion for the pairwise comparisons between Omental and intramuscular (OI), Omental and subcutaneous (OS), intramuscular and subcutaneous (IS), and intramuscular and the combined omental and subcutaneous (IC). After negative binomial models are fitted and dispersion estimate are obtained, a generalized linear model (GLM) likelihood ratio test was used to determine differential expression. The GML likelihood ratio test automatically takes all known sources of variation into account. Significant DEGs each pairwise comparison were selected at FDR<0.01. In order to investigate differentially regulated genes specific to each depot, we selected common DEGs from two-way pairwise comparisons with one of tissues in common. For example, we compared DEGs with the same direction of log FC between OI and OS and selected common DEG to identify the omental-specifically regulated genes. Same methods were applied to detect DEGs specific to the intramuscular and subcutaneous fats.

### Functional Annotation of DEGs

The bovine Ensembl gene IDs were converted to official gene symbols by cross-matching to human Ensembl gene IDs and official gene symbols. The official gene symbols of human homologues of bovine genes were used for functional clustering and enrichment analyses using the Database for Annotation, Visualization and Integrated Discovery (DAVID) [24]. The representation of functional groups in omental, intramuscular, and subcutaneous fat tissue relative to the whole genome was investigated using the Expression Analysis Systematic Explorer (EASE) tool [25] within DAVID, which is a modified Fisher’s exact test to measure enrichment of gene ontology (GO) terms [26]. To identify enrichemented GO terms functionally clustered genes were filtered by EASE value <0.01 and selected.

### Reverse Transcription and Quantitative Real-time PCR

The 1.5 ug of extracted total RNA was used in a final volume of 20 µl to synthesize cDNA using a High Capacity RNA-to-cDNA Kit (Applied Biosystems, Foster City, CA). With the synthesized cDNA sample of each tissue, quantitative real-time PCR was performed to analyze the quantity of mRNA that coded for the selected genes using the ABI 7300 Real-Time PCR system (Applied Biosystems, Foster City, CA) and a quantitative real-time PCR kit (DyNAmo HS SYBR Green qPCR kit, Finnzymes, Finland). Amplification was carried out for 1 cycle at 95°C for 5 min; 40 cycles at 95°C for 15 s, annealing temperature listed on the Table S1 for 30 s, 72°C for 30 s; 1 cycle of 72°C for 10 min. For amplification, 1 pM of the primers were used for the amplification. The mRNA quantities of the target genes were normalized to that of a reference gene. The qRT-PCR for all target genes was performed twice with two different reference genes, *β-actin* (*ACTB*) and *glyceraldehyde-3-phosphate dehydrogenase* (*GAPDH*), to make the result more reliable. Using threshold cycle (CT) values for these genes, relative expression levels were calculated with the 2^−ΔΔ^Ct method [27].

## Results

### Quality of RNA-sequence Reads in Adipose Tissues

We obtained RNA-seq reads from three different adipose tissues (subcutaneous, intramuscular, and omental) of nine Hanwoo animals [GenBank:GSE39618]. The numbers of total sequence reads and mapping rates for each sample were summarized in Table S2. Only a few amount of sequence reads (<0.05%) did not pass the quality filtering. The average numbers of sequence reads in each tissue were 38, 36, and 35 M reads in subcutaneous (S), intramuscular (I), and omental (O) fat respectively. Among the sequence reads passed the quality control, on average, 96.9% reads in subcutaneous fat, 98.3% in intramuscular, and 97.5% in omental fat were successfully mapped to bovine genome (bosTau6) using TopHat (v2.0.2).

### Identification of Differentially Expressed Genes (DEGs)

**Figure 1:**
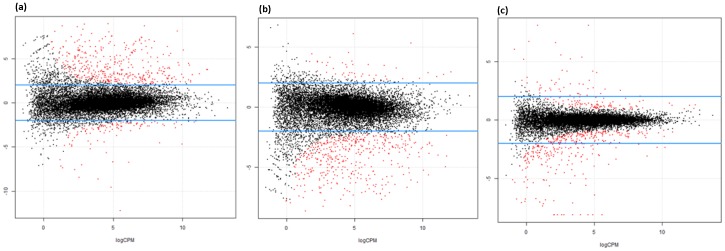
Cox-Reid common dispersion between adipose depots. (a) Omental vs. Intramuscular, (b) intramuscular vs. subcutaneous, and (c) omental vs. subcutaneous.

To investigate the difference in expression profiles of genes among adipose tissues of subcutaneous (S), intramuscular (I), and omental (O), we identified differentially expressed genes (DEGs) in the pairwise comparison of three adipose depots [28] (Figure 1). We found 5797 (3080 up-regulated in I and 2717 in O), 2156 (836 up-regulated in S and 1320 in O), and 5454 DEGs (2399 up-regulated in S and 3055 in I) in the comparison of omental and intramuscular fat (OI), the omental and subcutaneous fat (OS), and the intramuscular and subcutaneous fat (IS), respectively (FDR<0.01) (Table 1, File S1). The number of DEG in OI and IS were two times higher compared with the OS. We also compared between the intramuscular fat (I) and the combined omental and subcutaneous fat (C) and found 5657 DEGs (2992 up-regulated in I and 2665 in C) (Table 1, File S1), which showed the similar pattern when the intramuscular fat was compared with other adipose fats separately. In order to identify depot specific DEG, we selected common DEGs from the two-way pairwise comparisons with one of tissues in common. We found 853 (302 up-regulated and 551 down-regulated), 4276 (2507 up-regulated and 1769 down-regulated), and 979 (621 up-regulated and 1320 down-regulated) DEGs in subcutaneous (S), intramuscular (I), and omental (O) respectively (Table 1). The number of DEGs in the intramuscular was 4 times higher than that in other tissues.

**Table 1:** Summary of DEG identified from the comparison among three different adipose tissues (FDR<0.01).

Paired comparison				
	OS	OI	IS	IC
No. of DEG	2156	5797	5454	5657
up-regulated in (tissue)	836 (S)	3080 (I)	2399 (S)	2992 (I)
	1320 (O)	2717 (O)	3055 (I)	2665 (C)
**Tissue specific DEGs**				
	**Omental**	**Intramuscular**	**subcutaneous**	
	**(OS vs OI)**	**(IS vs IO)**	**(SI vs SO)**	
No. of DEG	979	4276	853	
up-regulated	621	2507	302	
down-regulated	358	1769	551	

### Validation of DEGs Using qRT-PCR

**Figure 2:**
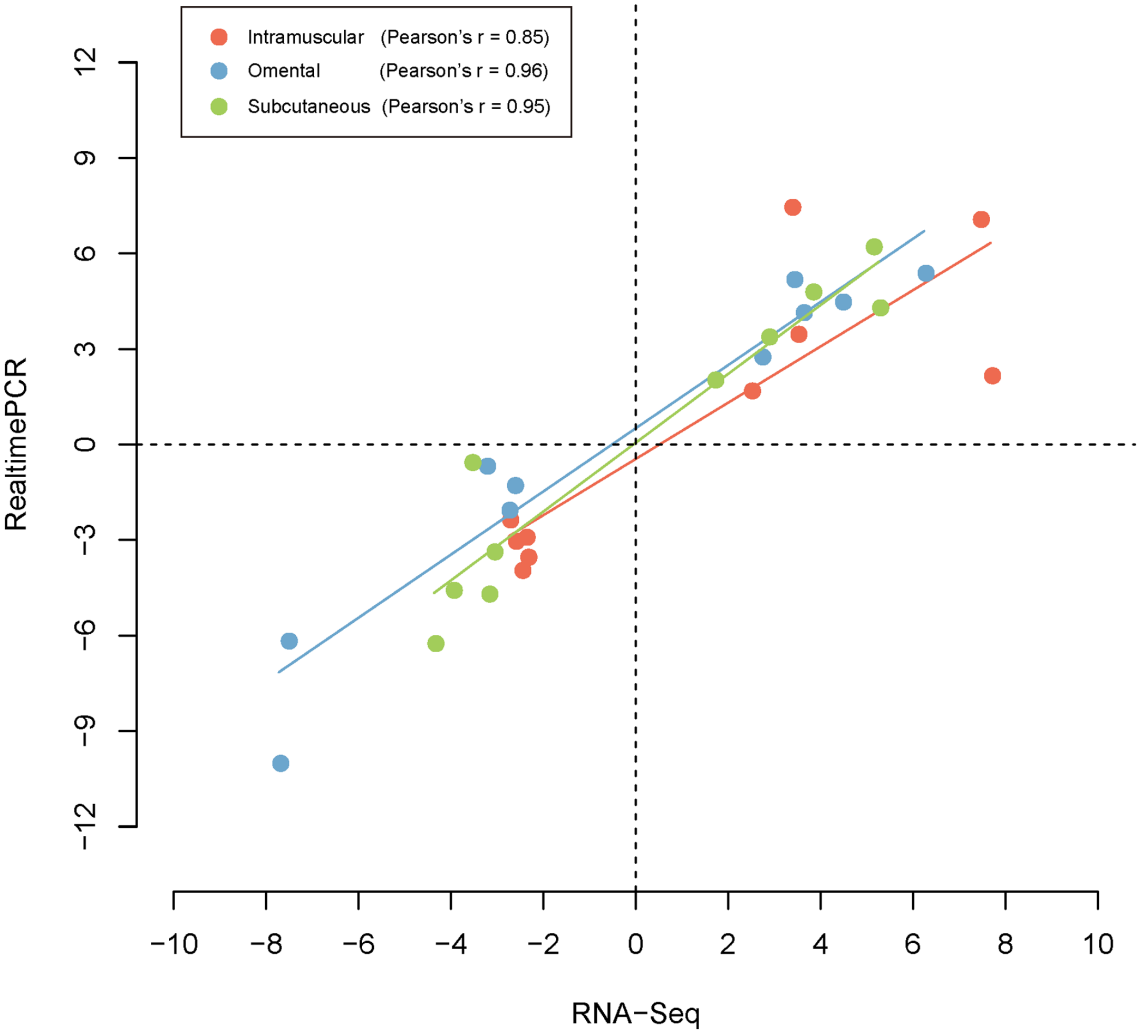
The correlation of fold changes in gene expression between the RNA seq and qRT-PCR.

We performed qRT-PCR to technically validate the DEGs detected in each adipose tissue. A total of 30 genes consisting of ten genes (5 up-regulated and 5 down-regulated) in each tissue were randomly selected with logFC>2 (Table S1). Correlation was calculated to compare the expression levels between RNAseq and qRT-PCR [27]. Fold changes of DEGs between two techniques were significantly correlated in three depots (I: Pearson’s r^2^ = 0.0,85, O: Pearson’s r = 0.96, and S: Pearson’s r = 0.95) (Figure 2, Table S3, and Table S4). The results confirmed that DEGs identified in this study were very reliable.

### DEGs Involved in the Adipogenesis and Lipid Metabolism

Literatures have reported that many genes were involved in the adipogenesis and lipid metabolism. Among the depot-specific DEGs identified, we found 28 genes related to the mechanisms (Table 2). *CEBPG*, *DDIT3*, *KLF7*, and *EBF1* that were known to function as anti-adipogenic factor increased in intramuscular fat. *UCP1*, *FOXC2*, and *PPARGC1A* related to brown adipogenesis were detected in the omental and intramuscular depots. *AIPOQ*, *FABP4*, and *FAS* that were involved in the fatty acid synthesis and metabolism were identified in intramuscular fat. *FASN*, *LPL*, and *DGAT1* associated with the determinants of the deposition of intramuscular fat were down-regulated in the intramuscular depot.

**Table 2:** Identified DEGs involving in adipogenesis, signaling pathway, and lipid metabolism.

Gene	Full Name	Tissues expressed	Reference
*ADIPOQ*	adiponectin, C1Q, and collagen domain containing	I(−)	Wang et al 2009 [22]
*BMP2*	bone morphogenetic proteins 2	O(−)	Rosen and MacDougald 2006 [63]
*BMP4*	bone morphogenetic proteins 4	S(−)	Rosen and MacDougald 2006 [63]
*CDK6*	cyclin-dependent kinase 6	I(+)	Sarruf et al 2005 [64]
*CEBPG*	CCAAT/enhancer binding protein (C/EBP), gamma	I(+)	Rosen and MacDougald 2006 [63]
*DDIT3*	DNA-damage-inducible transcipt 3 (CHOP)	I(+)	Darlington at al 1998 [65]
*DGAT1*	diacylglycerol acyltransferase	I(−)	Jeong et al 2012 [54]
*EBF1*	early B-cell factor 1	O(−)	Akerblad et al 2002 [66]
*EGR2*	early growth response 2	I(+), S(−)	Chen et al 2005 [67]
*FABP4*	fatty acid binding protein4	I(−)	Michal et al 2006 [68]
*FAS*	Fas (TNF receptor superfamily, member 6)	I(+)	Wang et al 2009 [22]
*FASN*	fatty acid synthase	I(−), S(+)	Jeong et al 2012 [54]
*FGF1*	fibroblast growth factor 1	O(+)	Hutley et al 2004 [69]
*FGF2*	fibroblast growth factor 2	S(−)	Kawaguchi et al 1997 [70]
*FOXC2*	forkhead box C2	I(+)	Rosen and MacDougald 2006 [63]
*GATA2*	GATA binding protein 2	I(+)	Tong et al 2000 [71]
*KLF5*	Kruppel-like factor 5	S(−), I(+)	Oishi et al 2005 [72]
*KLF6*	Kruppel-like factor 6	I(+), O(−)	Li et al 2005 [73]
*KLF7*	Kruppel-like factor 7	I(+)	Kanazawa et al 2005 [74]
*LPL*	lipoprotein lipase	O(+), I(−)	Jeong et al 2012 [54]
*PPARG*	peroxisome profiliferator-activated receptor, gamma	I(−)	Rosen and MacDougald 2006 [63]
*PPARGC1A*	peroxisome proliferator activated receptor gamma, coactivator 1 alpha	O(−)	Scime et al 2005 [75]
*THRSP*	thyroid hormone responsive	O(+), I(−)	Wang et al 2009 [22]
*UCP1*	uncoupling protein 1	O(+)	Rosen and MacDougald 2006 [63]
*UQCRC1*	ubiquinol-cytochrome c reductase core protein 1	I(−)	Kunej et al 2007 [76]
*WNT10B*	wingless-type MMTV integration site family, member 10B	O(−)	Longo et al 2004 [77]
*WNT5b*	wingless-type MMTV integration site 5B	S(−)	Kanazawa et al 2005 [78]
*WNT6*	wingless-type MMTV integration site 6	I(+)	Tseng et al 2005 [79]

(+) : up-regulated.

(−) : down-regulated.

### Gene Ontology and Functional Annotation of DEGs

**Figure 3:**
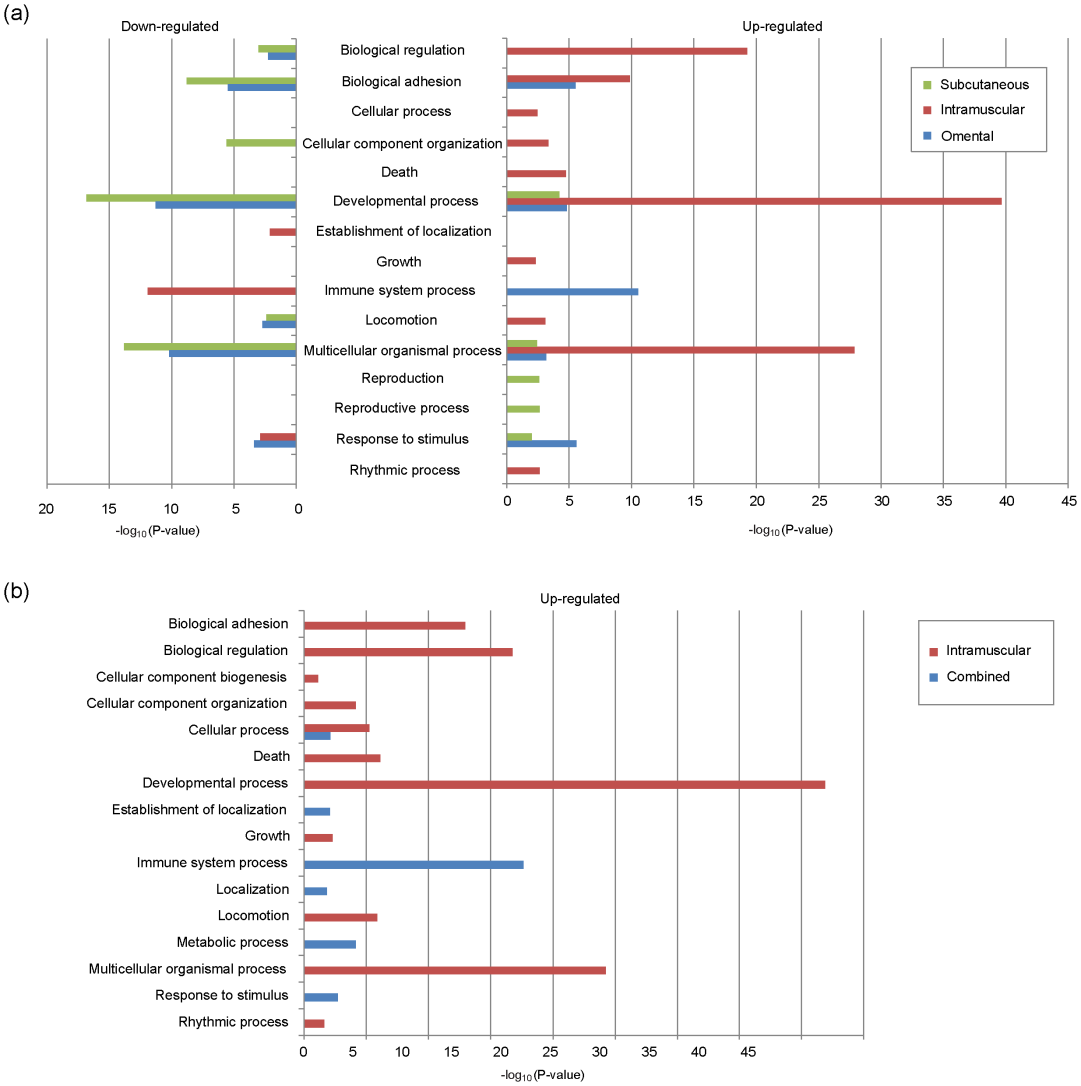
Biological process GO terms analysis of depot specifically regulated DEGs (a) and DEGs identified from the comparison between I and C (b).

Biological process gene ontology of DEGs from the pairwise comparison was summarized in Table S5. In intramuscular fat, developmental process was the most significantly enriched term compared to other two tissues (OI: p.value = 1.51e-47 and IS: p.value = 2.30e-49). Also developmental process was significantly enriched in the both omental (p.value = 9.47e-16) and subcutaneous fat (6.23e-12) when they were compared each other. Gene ontology (GO) analysis was also performed with the depot specific DEGs. Biological process GO terms of each adipose tissue were summarized in Figure 3a. Most significantly enriched 3 GO terms of DEGs up-regulated in the intramuscular fat were developmental process (p.value = 2.30e-40), multicellular organismal process (p.value = 1.40e-28), and biological regulation (p.value = 1.40e-10). Immune system process (p.value = 2.90e-11) and developmental process (p.value = 6.20e-05) were the most significantly enriched GO terms of DEGs up-regulated genes in the omental and subcutaneous fat respectively For the DEGs down-regulated in each depot, developmental process (S: p.value = 1.80e-17, O: p.value = 5.80e-12) and multicellular organismal process (S: p.value = 1.80e-14 O: p.value = 6.90e-11) were the most significantly enriched in both subcutaneous and omental fat. In the intramuscular fat, immune system process (p.value = 1.40e-12) was the most significant GO terms of biological process. When intramuscular fat was compared with the combined omental and subcutaneous fat, developmental process (p.value = 1.28e-42) and immune system process (p.value = 2.33e-18) were most significantly enriched in intramuscular and the combined respectively (Figure 3b). GO analysis of cellular components and molecular function were summarized in Table S6. Overall cellular component GO terms of depot-specific DEGs were related to the extracellular region and cell part, and molecular function GO terms were involved in the various molecule activity.

### KEGG Pathway Analysis of Depot Specific DEGs

**Figure 4:**
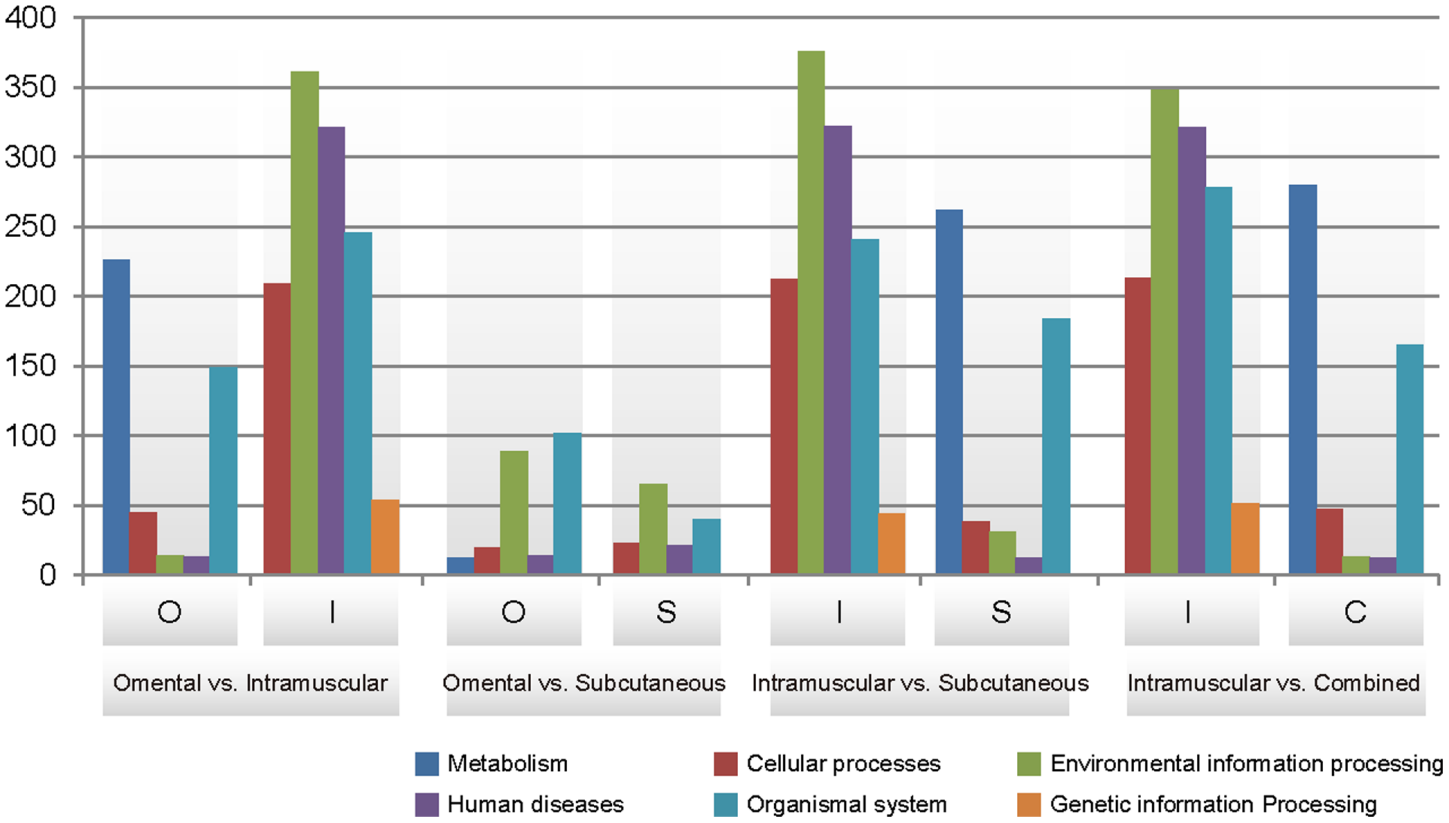
Representative KEGG pathways of DEGs resulted from the pairwise comparison among different adipose depots. Y-axis shows the number of gene counts.

KEGG pathway analysis of DEG resulted from the pairwise comparison showed that DEGs were largely clustered into 6 representative terms (higher level of the pathways) including metabolism, cellular processes, environmental information processing, human diseases, organismal system, and genetic information processing (Figure 4, and Table S7). Many DEGs in intramuscular fat were highly enriched in the pathways of environmental information processing. When the omental fat was compared to intramuscular fat (OI), the top significant pathways were valine, leucine and isoleucine degradation (p. value = 3.39E-11), PPAR signaling pathway (p.value = 8.91E-10), lysosome (p.value = 2.59E-09) and fatty acid metabolism (p.value = 7.38E-09) (Table S7 (a)). When the subcutaneous fat was compared to intramuscular fat (SI), similar pathways were observed with less significant p-value (Table S7 (b)). The most significant pathway in both comparisons is valine, leucin and isoleucine degradation which is an amino acid metabolism. KEGG pathways enriched in the intramuscular fat showed the similar pattern of the pathways when it was compared to omental and subcutaneous fats (Table S7 (c) and (d)). Ribosome (p.value = 1.15E-20), hypertrophic cardiomyopathy (p.value = 2.94E-15), dilated cardiomyopathy (p.value = 2.73E-14), and focal adhesion (p.value = 2.22E-10) were top significant pathways compared to other adipose with the little difference of p.vaules. PPAR signaling pathway (p.value = 2.84E-07) and ECM-receptor interaction (p.value = 4.65E-07) are the most enriched pathways in omental fat and subcutaneous fat respectively when they were compared each other (Table S7, (e) and (f)). When intramuscular fat (I) was compared to the combined omental and subcutaneous fats (C), same KEGG pathways were enriched in the intramuscular fat and in the combined fats as the intramuscular fat was compared to other fats separately (Table S7, (g) and (h)). KEGG pathway analysis of depot specific DEG identified 4 significantly enriched pathways of up-regulated genes in omental fat, 16 pathways in intramuscular fat, and 2 in subcutaneous fat tissue (p<0.01) (Table S8). In omental fat, DEGs related to lipid metabolisms were highly up-regulated, while those related to signaling modules and interactions were down-regulated. In intramuscular fat, DEGs involved in the human cardiac muscle disease such as cardiomyopathy (HCM, ARVC, and DCM), cancer, and Type II diabetes mellitus were most significantly up-regulated but those related to the lipid metabolism and immune response were down regulated. In subcutaneous fat, genes in the pathways of host defense were up-regulated and those involved in the disease and cancer were down –regulated.

### Developmental Process and ECM-receptor Interaction

**Figure 5:**
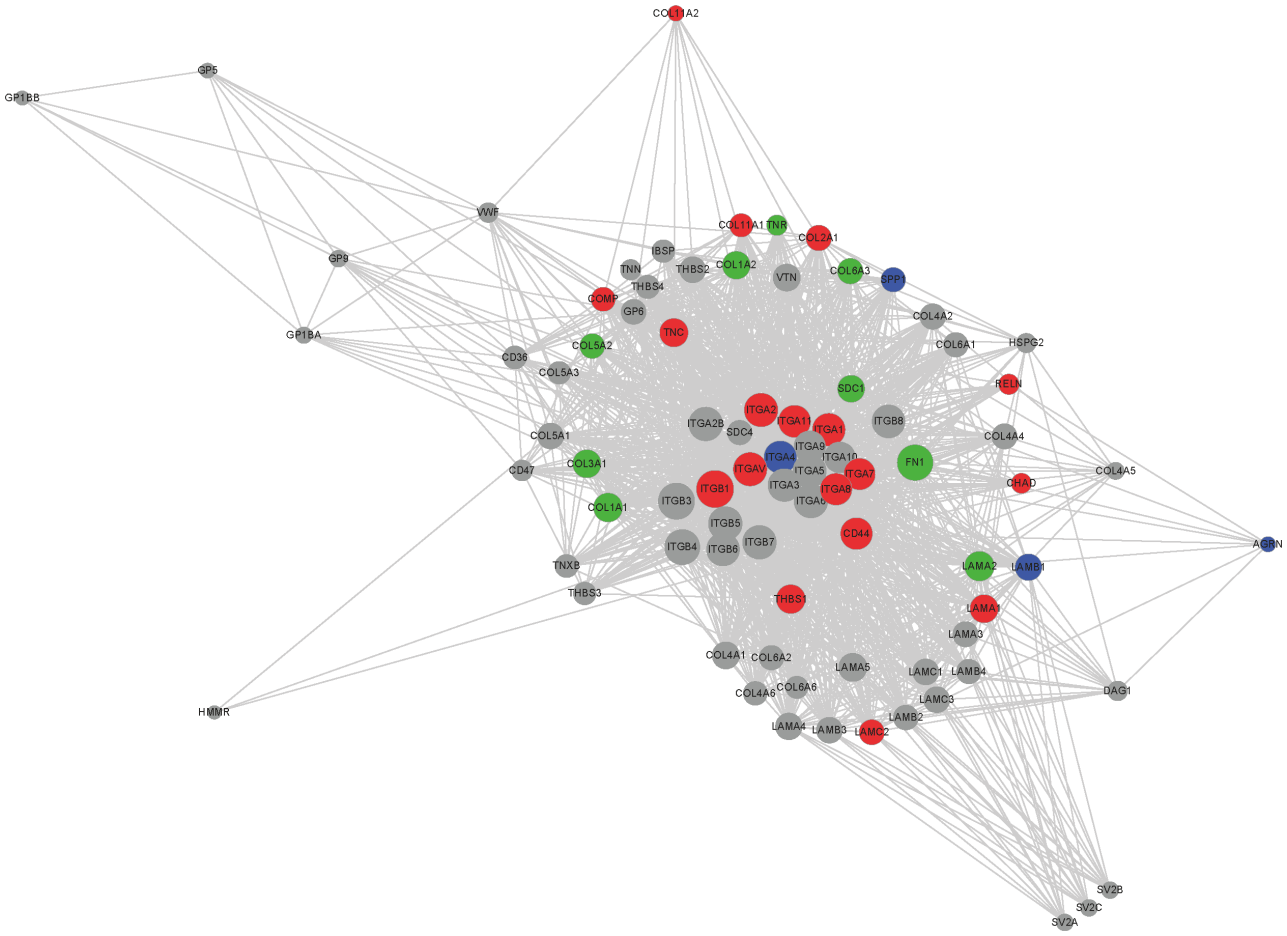
Visualization of molecular network in the KEGG pathway of ECM-receptor interaction using cytoscape. Grey circles represent genes involved in the pathway and the color of the circle represent genes highly expressed in each depot (red:intramuscular, blue: omental, green: subcutaneous).

Significant numbers of DEGs from the adipose tissues were enriched in various GO terms of the developmental process and their KEGG pathway analysis showed that several pathways including, axon guidance, pathways in cancer, and ECM-receptor interaction were common in all three fat tissues (Table 3). Among those pathways, interestingly, the ECM-receptor interaction also involved in other enriched pathways such as pathways in cancer, dilated cardiomyopathy, hypertrophic cardiomyopathy, and Arrhythmogenic right ventricular cardiomyopathy, which were the most significant in intramuscular fat. Although ECM-receptor interaction was common pathway in the three adipose tissues, genes involved in the pathways were differentially regulated (Figure 5). Among genes in ECM-receptor interaction, 9, 4, and 18 genes were significantly up-regulated in subcutaneous, omental and intramuscular fat respectively (Figure 5). Collagen genes (*COL1A1*, *COL1A2*, *COL3A1*, *COL5A2*, and *COL6A3*) were significantly up-regulated in subcutaneous fat and integrins (*ITGA1*, *2*, *7*, *8*, *11*, *V*, and *ITGB1*) were significantly up-regulated in intramuscular fat, with some of other collagens (*COL2A1*, *COL11A1*, and *COL11A2*). Different laminins were up-regulated in the different depots, *LAMA2* in subcutaneous, *LAMB1* in omental, and *LAMB3* in intramuscular fat.

**Table 3:** KEGG pathways of DEGs involved in developmental process.

Depot	KEGG ID	Terms	P-value
Omental	hsa04360	Axon guidance	3.25E-06
	hsa05200	Pathways in cancer	3.81E-04
	hsa05217	Basal cell carcinoma	8.91E-04
	hsa05340	Primary immunodeficiency	2.95E-03
	hsa04514	Cell adhesion molecules (CAMs)	4.37E-03
	hsa04512	ECM-receptor interaction	9.97E-03
subcutaneous	hsa04510	Focal adhesion	1.97E-05
	hsa04512	ECM-receptor interaction	2.39E-05
	hsa05200	Pathways in cancer	3.60E-04
	hsa04360	Axon guidance	6.61E-04
intramuscular	hsa05410	Hypertrophic cardiomyopathy (HCM)	1.24E-13
	hsa05414	Dilated cardiomyopathy	7.07E-12
	hsa05200	Pathways in cancer	1.19E-11
	hsa04510	Focal adhesion	5.76E-10
	hsa04512	ECM-receptor interaction	5.21E-07
	hsa04350	TGF-beta signaling pathway	8.81E-07
	hsa04360	Axon guidance	1.64E-05
	hsa05219	Bladder cancer	2.79E-05
	hsa05412	Arrhythmogenic right ventricular cardiomyopathy (ARVC)	7.71E-05
	hsa04010	MAPK signaling pathway	2.84E-04
	hsa04810	Regulation of actin cytoskeleton	3.64E-04
	hsa04630	Jak-STAT signaling pathway	6.07E-04
	hsa04060	Cytokine-cytokine receptor interaction	1.14E-03
	hsa04270	Vascular smooth muscle contraction	1.19E-03
	hsa04310	Wnt signaling pathway	1.22E-03
	hsa04115	p53 signaling pathway	1.76E-03
	hsa05222	Small cell lung cancer	2.73E-03
	hsa04340	Hedgehog signaling pathway	5.99E-03
	hsa04020	Calcium signaling pathway	6.18E-03
	hsa05210	Colorectal cancer	8.31E-03

## Discussion

The amount and distribution of fat are known to be important factors to influence the meat quality in the beef industry. Especially, intramuscular fat (marbling) plays important roles in eating quality and composition of meat compared to other fats [1], [2]. Some literatures reported that intramuscular fat is different from other fats in three respects: adipocyte size, metabolic activities, and developmental timing [29]–[31]. Intramuscular adipose tissue is a later developing tissue and behaves different from other adipose tissues in development of cellularity and metabolic capacity [31]. Intramuscular adipocytes are smaller than non-muscular adipocytes in cattle and pigs [29], [32]. Metabolic activities including lipogenesis and lipolysis in intramuscular adipocytes were lower than other adipocytes by proteomic analysis, gene expression and/or enzyme activity [29], [30]. Genetic mechanisms in intramuscular fat might be different from those in other fat depots. However little has been known about the genetic and functional difference of the intramuscular fat compared to others. In order to investigate the genetic profiles of fat tissues based on depots and understand the difference of the genetic mechanisms, we performed the transcriptome analysis from 3 different adipose tissues of omental, subcutaneous, and intramuscular fat using Illumina HiSeq 2000.

### Sequencing Quality and Coverage

We obtained RNA-seq transcriptome data from omental, subcutaneous and intramuscular fats of nine cattle. The transcriptome data generated from the NGS provided very reliable sequences reads because over 99% of the sequence reads passed the QC control and the mapping rate of the sequence reads to bovine genome was very high. Our data also showed that the sequencing depth is deep enough for this study. We obtained more than 30 M reads from most of samples. Although it might not be meaningful to mention the sequencing coverage of transcriptome, we would like to have an idea how well the depth of the sequence could cover the adipose transcriptome. There are several studies showing that about 30 M reads is sufficient to detect >90% of annotated genes in yeast, chicken, and cattle [33]–[35], which suggested that our data had enough coverage, even though the tissues used for the study were different.

### Different Genetic Profile in Intramuscular Fat

Identification of DEGs among the fat tissues from the 3 different adipose depots supported that the intramuscular fat had more different genetic profiles than both the omental and subcutaneous fats did. The numbers of DEG showed two times higher when the intramuscular fat was compared to other adipose tissues than when omental fat and subcutaneous fat were compared each other (Table 1). When the intramuscular fat was compared with the combined omental and subcutaneous fat, similar numbers of DEG were identified. This also confirmed that omental and subcutaneous fat shared many adipogenetic genes and pathways compared to that in intramuscular depot. The functional annotation analysis also presented the similar GO biological process terms and KEGG pathways in the omental and subcutaneous fats, but not in intramuscular fat (Table S5, Table S7, Table S8, Figure 3 and Figure 4), where DEGs in pathways of lipid metabolisms such as PPAR signaling pathways and fatty acid metabolism, and of the immune response systems were significantly down regulated.

### Lower Metabolic Activities of Intramuscular Fat

One of the significantly enriched pathways of up-regulated genes in the omental fat was PPAR signaling pathway. Recent studies proved that PPARs functions as a key regulator of adipocyte differentiation, accumulation and phenotypes [36]. Genes enriched in the pathway are involved in lipid metabolisms including fatty acid transport (*LPL*, *ACSL1*, and *OLR1*), fatty acid oxidation (*EHHADH*, *CPT1B*, *ACADL*, and *ACADM*), adaptive thermogenesis (*UCP1*), and gluconeogenesis (*PCK1*) [37]–[50]. Most of them were also involved in the fatty acid metabolism pathway. PPARs (peroxisome proliferators-activated receptors) are known to play a key role in the metabolic pathways involving fatty acid oxidation and lipid metabolism [51]. We found 28 genes known to be involved in the adipogenesis and lipid metabolism in the previous studies (Table 2). Based on the expression patterns, many of them were significantly down-regulated in the intramuscular fat compared to other tissues. *PPARG*, a master regulator of adipogenesis, was down-regulated and other anti-adipogenic genes (*CEBPG*, *DDIT3*, *GATA2*, and *KLF7*) were up-regulated in intramuscular. Our results in part supported that intramuscular adipocytes had the low metabolic activities related to lipid metabolism based on transcriptome analysis with more various range of metabolic pathways and genes. Some genes involved in intramuscular deposition (*FASN*, *FABP4*, *LPL*, *THRSP*, and *DGAT1*) were also down-regulated in intramuscular fat. They were highly expressed in the early growth of cattle with high starch feeding and showed significant positive correlation with intramuscular fat content [52]–[54]. It seems that the expression results of those genes in this study were different from the previous. However it is difficult to simply compare those results because our study doesn’t have information of intramuscular fat deposition. Further study might need to clear out if the difference of gene expression was caused by the age difference because animals used in this study are over 30 month old or if the expression of the genes in intramuscular fat was relatively lower because our analysis was based on the direct comparisons between different fat tissues. In addition, it might be more efficient to identify the functional effect of intramuscular fat deposition on meat quality with different tissue sampling of intramuscular fats which was grouped based on the meat quality indices such as intramuscular fat contents and water contents.

### Pathways of Interested in each Adipose Depot

There were depot specifically enriched pathways in each adipose depot (Table S8). In omental fat, several members of CLDN family were differentially expressed in the pathways of leukocyte transendotheilial migration (*CLDN1*, *3*, and *15*), and cell adhesion molecules (*CLDN5*, and *10*). The claudin (CLDN) family plays a major role in tight junction-specific obliteration of the intercellular space through calcium-independent dell-adhesion activity. *CLDN6* is known to be an important regulator in adipogenesis and fat deposition but is not detected in this study [55]. Genetic manipulation of *CLDN1* expression induced changes in cellular phenotype [56]. This family might affect the depot-specific morphology of adipocytes by regulating tight junctions between cells. DEGs down-regulated in the subcutaneous fat were significantly enriched in pathway of melanogenesis. A recent study demonstrated that melanic biosynthesis pathway was functional in adipose tissues, and melanin and other proteins in the melanogenic pathway of adipocytes were expressed in higher level in the obese [57]. WNT signaling in the melanogenesis pathway is an important regulator for adipogenesis or insulin secretion. Expression of *WNT5B* gene was increased at an early phase of adipocyte differentiation in mouse and involved in the pathogenesis of the type 2 diabetes through the regulation of adipocyte function [58]. It was interesting that the cardiovascular disease related pathways (dilated cardiomyopathy, hypertrophic cardiomyopathy, and Arrhythmogenic right ventricular cardiomyopathy) were most significantly enriched in the intramuscular fat. There is no direct evidence showing that fat deposition in skeletal muscle caused the cardiac muscle diseases. However, excess body weight caused by chronic-over nutrition and high-fat feeding was associated with metabolic and/or diabetic cardiomyopathy in human [59].

### ECM-receptor Interaction and Differentially Regulated Genes Based on Adipose Depot

Based on the functional annotation analysis of DEGs from different adipose tissue, we identified the ECM-receptor interaction pathway was involved in the various pathways of the three adipose tissues. The extracellular matrix (ECM) is essential for tissue architecture and has an important role in adipogenesis [60]. However it has not received sufficient attention in adipose tissue because of the difficulties associated with analysis of ECM components. ECM consists of a complex mixture of structural and functional macromolecules including glycosaminoglycans (GAGs) and fibrous proteins (collagen, elastin, fibronection, and lammin) [60]. The main constituents of ECM in adipose tissue are collagen (type I, IV, and VI), laminin (LN1,8), fibronectin (FN), hyaluronan, and proteoglycan [61]. Specific interactions between cells and the ECM are mediated by transmembrane molecules such as integrins. In this study, different members of ECM fibrous proteins and integrins were up-regulated in the different adipose tissues. Collagens and integrins were significantly enriched in subcutaneous and intramuscular fat respectively. It seems that the interactions between ECM components and integrins were differentially regulated based on the adipose depots. Several studies reported that members of integrins showed the expression change associated with adipocyte differentiation [62]. Other genes involved in the ECM–receptor interaction were also showed that they were differentially regulated depending on the adipose depots. This comparative transcriptome analysis of 3 adipose tissues showed that the interaction between ECM components and transmembrane receptors of fat cells might influence the depot specific adipogenesis.

## Supporting Information

Figure S1 **Data quality control using FastQC.**
(DOCX)

Table S1 The primer sequence of DEG used for qRT-PCR analysis.
(DOCX)

Table S2 **RNA-seq reads and mapping rate of different adipose depots from nine Hanwoo individuals.**
(DOCX)

Table S3 **Quantitative RT-PCR validation of DEGs in each adipose tissue.**
(DOCX)

Table S4 **Statistical analysis of the correlation between RNAseq and qRT-PCR.**
(DOCX)

Table S5 **Biological process GO terms of DEGs resulted from pairwise comparison among three different adipose depots.**
(DOCX)

Table S6 **GO terms of cellular components and molecular function of depot specific DEGs.**
(DOCX)

Table S7 **KEGG pathways of DEGs resulted from the pairwise comparison among three different adipose depots.**
(DOCX)

Table S8 **Summary of the enriched KEGG pathway results based on the depot specific DEGs.**
(DOCX)

File S1 **DEG lists based on the comparison between adipose depots.**
(XLSX)

## References

[1] Brooks MA, Choi CW, Lunt DK, Kawachi H, Smith SB (2011) Subcutaneous and intramuscular adipose tissue stearoyl-coenzyme A desaturase gene expression and fatty acid composition in calf- and yearling-fed Angus steers. J Anim Sci 89: 2556–2570. 21454869 10.2527/jas.2010-3369

[2] Dodson MV, Jiang Z, Chen J, Hausman GJ, Guan le L, et al (2010) Allied industry approaches to alter intramuscular fat content and composition in beef animals. J Food Sci 75: R1–8. 20492190 10.1111/j.1750-3841.2009.01396.x

[3] Arner P (1997) Regional adipocity in man. J Endocrinol 155: 191–192. 9415044 10.1677/joe.0.1550191

[4] Kirkland JL, Cummins P, Steppan C, Dobson DE, Cladaras MH (1997) Decreasing preadipocyte differentiation capacity with aging is associated with blunted expression of the transcription factor, CCAAT enhancer binding protein a. Obes Res 5: 22S.

[5] Kirkland JL, Dax EM (1984) Adipocyte hormone responsiveness and aging in the rat: problems in the interpretation of aging research. J Am Geriatr Soc 32: 219–228. 6321584 10.1111/j.1532-5415.1984.tb02006.x

[6] Billon N, Monteiro MC, Dani C (2008) Developmental origin of adipocytes: new insights into a pending question. Biol Cell 100: 563–575. 18793119 10.1042/BC20080011

[7] Joe AW, Yi L, Even Y, Vogl AW, Rossi FM (2009) Depot-specific differences in adipogenic progenitor abundance and proliferative response to high-fat diet. Stem Cells 27: 2563–2570. 19658193 10.1002/stem.190

[8] Toyoda M, Matsubara Y, Lin K, Sugimachi K, Furue M (2009) Characterization and comparison of adipose tissue-derived cells from human subcutaneous and omental adipose tissues. Cell Biochem Funct 27: 440–447. 19691084 10.1002/cbf.1591

[9] Caserta F, Tchkonia T, Civelek VN, Prentki M, Brown NF, et al (2001) Fat depot origin affects fatty acid handling in cultured rat and human preadipocytes. Am J Physiol Endocrinol Metab 280: E238–247. 11158926 10.1152/ajpendo.2001.280.2.E238

[10] Edens NK, Fried SK, Kral JG, Hirsch J, Leibel RL (1993) In vitro lipid synthesis in human adipose tissue from three abdominal sites. Am J Physiol 265: E374–379. 8214046 10.1152/ajpendo.1993.265.3.E374

[11] Fried SK, Russell CD, Grauso NL, Brolin RE (1993) Lipoprotein lipase regulation by insulin and glucocorticoid in subcutaneous and omental adipose tissues of obese women and men. J Clin Invest 92: 2191–2198. 8227334 10.1172/JCI116821 PMC288398

[12] Hartman AD (1985) Adipocyte fatty acid mobilization in vivo: effects of age and anatomical location. Lipids 20: 255–261. 4010482 10.1007/BF02534256

[13] Hube F, Lietz U, Igel M, Jensen PB, Tornqvist H, et al (1996) Difference in leptin mRNA levels between omental and subcutaneous abdominal adipose tissue from obese humans. Horm Metab Res 28: 690–693. 9013743 10.1055/s-2007-979879

[14] Adams M, Reginato MJ, Shao D, Lazar MA, Chatterjee VK (1997) Transcriptional activation by peroxisome proliferator-activated receptor gamma is inhibited by phosphorylation at a consensus mitogen-activated protein kinase site. J Biol Chem 272: 5128–5132. 9030579 10.1074/jbc.272.8.5128

[15] Djian P, Roncari AK, Hollenberg CH (1983) Influence of anatomic site and age on the replication and differentiation of rat adipocyte precursors in culture. J Clin Invest 72: 1200–1208. 6630508 10.1172/JCI111075 PMC370403

[16] Hauner H, Entenmann G (1991) Regional variation of adipose differentiation in cultured stromal-vascular cells from the abdominal and femoral adipose tissue of obese women. Int J Obes 15: 121–126. 2040549

[17] Kirkland JL, Hollenberg CH, Gillon WS (1996) Effects of fat depot site on differentiation-dependent gene expression in rat preadipocytes. Int J Obes Relat Metab Disord 20 Suppl 3S102–107. 8680469

[18] Niesler CU, Siddle K, Prins JB (1998) Human preadipocytes display a depot-specific susceptibility to apoptosis. Diabetes 47: 1365–1368. 9703343 10.2337/diab.47.8.1365

[19] Hocquette JF, Gondret F, Baeza E, Medale F, Jurie C, et al (2010) Intramuscular fat content in meat-producing animals: development, genetic and nutritional control, and identification of putative markers. Animal 4: 303–319. 22443885 10.1017/S1751731109991091

[20] Basu U, Romao JM, Guan LL (2012) Adipogenic Transcriptome Profiling Using High Throughput Technologies Journal of Genomics. 1: 22–28. 10.7150/jgen.3781 PMC4091434 25031652

[21] Jin W, Olson EN, Moore SS, Basarab JA, Basu U, et al (2012) Transcriptome analysis of subcutaneous adipose tissues in beef cattle using 3' digital gene expression-tag profiling. J Anim Sci 90: 171–183. 21856901 10.2527/jas.2011-4229

[22] Wang YH, Bower NI, Reverter A, Tan SH, De Jager N, et al (2009) Gene expression patterns during intramuscular fat development in cattle. J Anim Sci 87: 119–130. 18820161 10.2527/jas.2008-1082

[23] Robinson MD, McCarthy DJ, Smyth GK (2010) edgeR: a Bioconductor package for differential expression analysis of digital gene expression data. Bioinformatics 26: 139–140. 19910308 10.1093/bioinformatics/btp616 PMC2796818

[24] Dennis Jr G, Sherman BT, Hosack DA, Yang J, Gao W, et al (2003) DAVID: database for annotation, visualization, and integrated discovery. Genome Biol 4: P3. 12734009 PMC193660

[25] Hosack DA, Dennis Jr G, Sherman BT, Lane HC, Lempicki RA (2003) Identifying biological themes within lists of genes with EASE. Genome Biol 4: R70. 14519205 10.1186/gb-2003-4-10-r70 PMC328459

[26] Alterovitz G, Ramoni MF (2010) Knowledge based bioinformatics: Wiley Online Library.

[27] Livak KJ, Schmittgen TD (2001) Analysis of relative gene expression data using real-time quantitative PCR and the 2(−Delta Delta C(T)) Method. Methods 25: 402–408. 11846609 10.1006/meth.2001.1262

[28] Robinson MD, Smyth GK (2008) Small-sample estimation of negative binomial dispersion, with applications to SAGE data. Biostatistics 9: 321–332. 17728317 10.1093/biostatistics/kxm030

[29] Gardan D, Gondret F, Louveau I (2006) Lipid metabolism and secretory function of porcine intramuscular adipocytes compared with subcutaneous and perirenal adipocytes. Am J Physiol Endocrinol Metab 291: E372–380. 16705057 10.1152/ajpendo.00482.2005

[30] Gondret F, Guitton N, Guillerm-Regost C, Louveau I (2008) Regional differences in porcine adipocytes isolated from skeletal muscle and adipose tissues as identified by a proteomic approach. Journal of Animal Science 86: 2115–2125. 18310487 10.2527/jas.2007-0750

[31] Lee YB, Kauffman RG (1974) Cellular and Enzymatic Changes with Animal Growth in Porcine Intramuscular Adipose Tissue. Journal of Animal Science 38: 532–537. 4819545 10.2527/jas1974.383532x

[32] Miller MF, Cross HR, Lunt DK, Smith SB (1991) Lipogenesis in acute and 48-hour cultures of bovine intramuscular and subcutaneous adipose tissue explants. J Anim Sci 69: 162–170. 2005009 10.2527/1991.691162x

[33] Li RW, Rinaldi M, Capuco AV (2011) Characterization of the abomasal transcriptome for mechanisms of resistance to gastrointestinal nematodes in cattle. Vet Res 42: 114. 22129081 10.1186/1297-9716-42-114 PMC3260172

[34] Nagalakshmi U, Wang Z, Waern K, Shou C, Raha D, et al (2008) The transcriptional landscape of the yeast genome defined by RNA sequencing. Science 320: 1344–1349. 18451266 10.1126/science.1158441 PMC2951732

[35] Wang Y, Ghaffari N, Johnson CD, Braga-Neto UM, Wang H, et al (2011) Evaluation of the coverage and depth of transcriptome by RNA-Seq in chickens. BMC Bioinformatics 12 Suppl 10S5. 10.1186/1471-2105-12-S10-S5 PMC3236848 22165852

[36] Cipolletta D, Feuerer M, Li A, Kamei N, Lee J, et al (2012) PPAR-gamma is a major driver of the accumulation and phenotype of adipose tissue Treg cells. Nature 486: 549–553. 22722857 10.1038/nature11132 PMC3387339

[37] Beale EG, Harvey BJ, Forest C (2007) PCK1 and PCK2 as candidate diabetes and obesity genes. Cell Biochem Biophys 48: 89–95. 17709878 10.1007/s12013-007-0025-6

[38] Chui PC, Guan HP, Lehrke M, Lazar MA (2005) PPARgamma regulates adipocyte cholesterol metabolism via oxidized LDL receptor 1. J Clin Invest 115: 2244–2256. 16007265 10.1172/JCI24130 PMC1172230

[39] Dhar M, Sepkovic DW, Hirani V, Magnusson RP, Lasker JM (2008) Omega oxidation of 3-hydroxy fatty acids by the human CYP4F gene subfamily enzyme CYP4F11. J Lipid Res 49: 612–624. 18065749 10.1194/jlr.M700450-JLR200

[40] Ditlecadet D, Short CE, Driedzic WR (2011) Glycerol loss to water exceeds glycerol catabolism via glycerol kinase in freeze-resistant rainbow smelt (Osmerus mordax). Am J Physiol Regul Integr Comp Physiol 300: R674–684. 21178128 10.1152/ajpregu.00700.2010

[41] Dube E, Gravel A, Martin C, Desparois G, Moussa I, et al.. (2012) Modulation of Fatty Acid Transport and Metabolism by Obesity in the Human Full-Term Placenta. Biol Reprod. 10.1095/biolreprod.111.098095 22553224

[42] Fisher FM, Kleiner S, Douris N, Fox EC, Mepani RJ, et al (2012) FGF21 regulates PGC-1alpha and browning of white adipose tissues in adaptive thermogenesis. Genes Dev 26: 271–281. 22302939 10.1101/gad.177857.111 PMC3278894

[43] Helledie T, Jorgensen C, Antonius M, Krogsdam AM, Kratchmarova I, et al (2002) Role of adipocyte lipid-binding protein (ALBP) and acyl-coA binding protein (ACBP) in PPAR-mediated transactivation. Mol Cell Biochem 239: 157–164. 12479581

[44] Houten SM, Denis S, Argmann CA, Jia Y, Ferdinandusse S, et al (2012) Peroxisomal L-bifunctional enzyme (Ehhadh) is essential for the production of medium-chain dicarboxylic acids. J Lipid Res 53: 1296–1303. 22534643 10.1194/jlr.M024463 PMC3371241

[45] Huang H, Atshaves BP, Frolov A, Kier AB, Schroeder F (2005) Acyl-coenzyme A binding protein expression alters liver fatty acyl-coenzyme A metabolism. Biochemistry 44: 10282–10297. 16042405 10.1021/bi0477891

[46] Li P, Li XB, Fu SX, Wu CC, Wang XX, et al (2012) Alterations of fatty acid beta-oxidation capability in the liver of ketotic cows. J Dairy Sci 95: 1759–1766. 22459824 10.3168/jds.2011-4580

[47] Lobo S, Bernlohr DA (2007) Fatty acid transport in adipocytes and the development of insulin resistance. Novartis Found Symp 286: 113–121; discussion 121–116, 162–113, 196–203. 10.1002/9780470985571.ch10 18269178

[48] Radler U, Stangl H, Lechner S, Lienbacher G, Krepp R, et al (2011) A combination of (omega-3) polyunsaturated fatty acids, polyphenols and L-carnitine reduces the plasma lipid levels and increases the expression of genes involved in fatty acid oxidation in human peripheral blood mononuclear cells and HepG2 cells. Ann Nutr Metab 58: 133–140. 21540583 10.1159/000327150

[49] Schreiber SN, Knutti D, Brogli K, Uhlmann T, Kralli A (2003) The transcriptional coactivator PGC-1 regulates the expression and activity of the orphan nuclear receptor estrogen-related receptor alpha (ERRalpha). J Biol Chem 278: 9013–9018. 12522104 10.1074/jbc.M212923200

[50] Yang Y, Eggertsen G, Gafvels M, Andersson U, Einarsson C, et al (2004) Mechanisms of cholesterol and sterol regulatory element binding protein regulation of the sterol 12alpha-hydroxylase gene (CYP8B1). Biochem Biophys Res Commun 320: 1204–1210. 15249218 10.1016/j.bbrc.2004.06.069

[51] Ament Z, Masoodi M, Griffin JL (2012) Applications of metabolomics for understanding the action of peroxisome proliferator-activated receptors (PPARs) in diabetes, obesity and cancer. Genome Med 4: 32. 22546357 10.1186/gm331 PMC3446260

[52] Graugnard D, Piantoni P, Bionaz M, Berger L, Faulkner D, et al (2009) Adipogenic and energy metabolism gene networks in longissimus lumborum during rapid post-weaning growth in Angus and Angus x Simmental cattle fed high-starch or low-starch diets. BMC Genomics 10: 142. 19335898 10.1186/1471-2164-10-142 PMC2676302

[53] Graugnard DE, Berger LL, Faulkner DB, Loor JJ (2010) High-starch diets induce precocious adipogenic gene network up-regulation in longissimus lumborum of early-weaned Angus cattle. Br J Nutr 103: 953–963. 20021700 10.1017/S0007114509992789

[54] Jeong J, Kwon EG, Im SK, Seo KS, Baik M (2012) Expression of fat deposition and fat removal genes is associated with intramuscular fat content in longissimus dorsi muscle of Korean cattle steers. J Anim Sci 90: 2044–2053. 22266990 10.2527/jas.2011-4753

[55] Hong YH, Hishikawa D, Miyahara H, Nishimura Y, Tsuzuki H, et al (2005) Up-regulation of the claudin-6 gene in adipogenesis. Biosci Biotechnol Biochem 69: 2117–2121. 16306693 10.1271/bbb.69.2117

[56] Dhawan P, Singh AB, Deane NG, No Y, Shiou SR, et al (2005) Claudin-1 regulates cellular transformation and metastatic behavior in colon cancer. J Clin Invest 115: 1765–1776. 15965503 10.1172/JCI24543 PMC1150288

[57] Randhawa M, Huff T, Valencia JC, Younossi Z, Chandhoke V, et al (2009) Evidence for the ectopic synthesis of melanin in human adipose tissue. FASEB J 23: 835–843. 18971261 10.1096/fj.08-116327 PMC2653983

[58] Kanazawa A, Tsukada S, Sekine A, Tsunoda T, Takahashi A, et al (2004) Association of the gene encoding wingless-type mammary tumor virus integration-site family member 5B (WNT5B) with type 2 diabetes. Am J Hum Genet 75: 832–843. 15386214 10.1086/425340 PMC1182112

[59] Mandavia CH, Pulakat L, DeMarco V, Sowers JR (2012) Over-nutrition and metabolic cardiomyopathy. Metabolism: clinical and experimental 61: 1205–1210. 22465089 10.1016/j.metabol.2012.02.013 PMC3393834

[60] Mariman EC, Wang P (2010) Adipocyte extracellular matrix composition, dynamics and role in obesity. Cell Mol Life Sci 67: 1277–1292. 20107860 10.1007/s00018-010-0263-4 PMC2839497

[61] Khan T, Muise ES, Iyengar P, Wang ZV, Chandalia M, et al (2009) Metabolic Dysregulation and Adipose Tissue Fibrosis: Role of Collagen VI. Molecular and Cellular Biology 29: 1575–1591. 19114551 10.1128/MCB.01300-08 PMC2648231

[62] Liu J, DeYoung SM, Zhang M, Zhang M, Cheng A, et al (2005) Changes in integrin expression during adipocyte differentiation. Cell Metab 2: 165–177. 16154099 10.1016/j.cmet.2005.08.006

[63] Rosen ED, MacDougald OA (2006) Adipocyte differentiation from the inside out. Nat Rev Mol Cell Biol 7: 885–896. 17139329 10.1038/nrm2066

[64] Sarruf DA, Iankova I, Abella A, Assou S, Miard S, et al (2005) Cyclin D3 Promotes Adipogenesis through Activation of Peroxisome Proliferator-Activated Receptor γ. Molecular and Cellular Biology 25: 9985–9995. 16260612 10.1128/MCB.25.22.9985-9995.2005 PMC1280250

[65] Darlington GJ, Ross SE, MacDougald OA (1998) The Role of C/EBP Genes in Adipocyte Differentiation. Journal of Biological Chemistry 273: 30057–30060. 9804754 10.1074/jbc.273.46.30057

[66] Åkerblad P, Lind U, Liberg D, Bamberg K, Sigvardsson M (2002) Early B-Cell Factor (O/E-1) Is a Promoter of Adipogenesis and Involved in Control of Genes Important for Terminal Adipocyte Differentiation. Molecular and Cellular Biology 22: 8015–8025. 12391167 10.1128/MCB.22.22.8015-8025.2002 PMC134715

[67] Chen Z, Torrens JI, Anand A, Spiegelman BM, Friedman JM (2005) Krox20 stimulates adipogenesis via C/EBPβ-dependent and -independent mechanisms. Cell Metab 1: 93–106. 16054051 10.1016/j.cmet.2004.12.009

[68] Michal JJ, Zhang ZW, Gaskins CT, Jiang Z (2006) The bovine fatty acid binding protein 4 gene is significantly associated with marbling and subcutaneous fat depth in Wagyu x Limousin F2 crosses. Anim Genet 37: 400–402. 16879357 10.1111/j.1365-2052.2006.01464.x

[69] Hutley L, Shurety W, Newell F, McGeary R, Pelton N, et al (2004) Fibroblast Growth Factor 1: A Key Regulator of Human Adipogenesis. Diabetes 53: 3097–3106. 15561939 10.2337/diabetes.53.12.3097

[70] Kawaguchi N, Toriyama K, Nicodemou-Lena E, Inou K, Torii S, et al (1998) De novo adipogenesis in mice at the site of injection of basement membrane and basic fibroblast growth factor. Proceedings of the National Academy of Sciences 95: 1062–1066. 10.1073/pnas.95.3.1062 PMC18672 9448285

[71] Tong Q, Dalgin G, Xu H, Ting C-N, Leiden JM, et al (2000) Function of GATA Transcription Factors in Preadipocyte-Adipocyte Transition. Science 290: 134–138. 11021798 10.1126/science.290.5489.134

[72] Oishi Y, Manabe I, Tobe K, Tsushima K, Shindo T, et al (2005) Krüppel-like transcription factor KLF5 is a key regulator of adipocyte differentiation. Cell Metab 1: 27–39. 16054042 10.1016/j.cmet.2004.11.005

[73] Li D, Yea S, Li S, Chen Z, Narla G, et al (2005) Krüppel-like Factor-6 Promotes Preadipocyte Differentiation through Histone Deacetylase 3-dependent Repression of DLK1. Journal of Biological Chemistry 280: 26941–26952. 15917248 10.1074/jbc.M500463200

[74] Kanazawa A, Kawamura Y, Sekine A, Iida A, Tsunoda T, et al (2005) Single nucleotide polymorphisms in the gene encoding Krüppel-like factor 7 are associated with type 2 diabetes. Diabetologia 48: 1315–1322. 15937668 10.1007/s00125-005-1797-0

[75] Scimè A, Grenier G, Huh MS, Gillespie MA, Bevilacqua L, et al (2005) Rb and p107 regulate preadipocyte differentiation into white versus brown fat through repression of PGC-1α. Cell Metab 2: 283–295. 16271529 10.1016/j.cmet.2005.10.002

[76] Kunej T, Wang Z, Michal JJ, Daniels TF, Magnuson NS, et al (2007) Functional UQCRC1 Polymorphisms Affect Promoter Activity and Body Lipid Accumulation[ast][ast]. Obesity 15: 2896–2901. 18198295 10.1038/oby.2007.344

[77] Longo KA, Wright WS, Kang S, Gerin I, Chiang S-H, et al (2004) Wnt10b Inhibits Development of White and Brown Adipose Tissues. Journal of Biological Chemistry 279: 35503–35509. 15190075 10.1074/jbc.M402937200

[78] Kanazawa A, Tsukada S, Kamiyama M, Yanagimoto T, Nakajima M, et al (2005) Wnt5b partially inhibits canonical Wnt/β-catenin signaling pathway and promotes adipogenesis in 3T3-L1 preadipocytes. Biochem Biophys Res Commun 330: 505–510. 15796911 10.1016/j.bbrc.2005.03.007

[79] Tseng Y-H, Butte AJ, Kokkotou E, Yechoor VK, Taniguchi CM, et al (2005) Prediction of preadipocyte differentiation by gene expression reveals role of insulin receptor substrates and necdin. Nat Cell Biol 7: 601–611. 15895078 10.1038/ncb1259

