# Supplementary figures and images for: Comparative Transcriptome Analysis of Adipose Tissues Reveals that ECM-Receptor Interaction Is Involved in the Depot-Specific Adipogenesis in Cattle
Source: PLoS One. 2013 Jun 21;8(6):e66267. doi: 10.1371/journal.pone.0066267 (PMC3689780; doi:10.1371/journal.pone.0066267)

**Figure S1. Data Quality Control using FastQC.**

1. **Per base sequence quality**


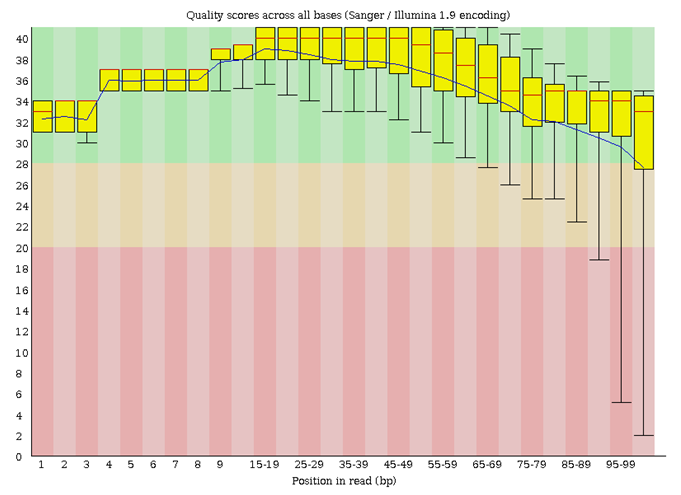


1. **Per base N content**


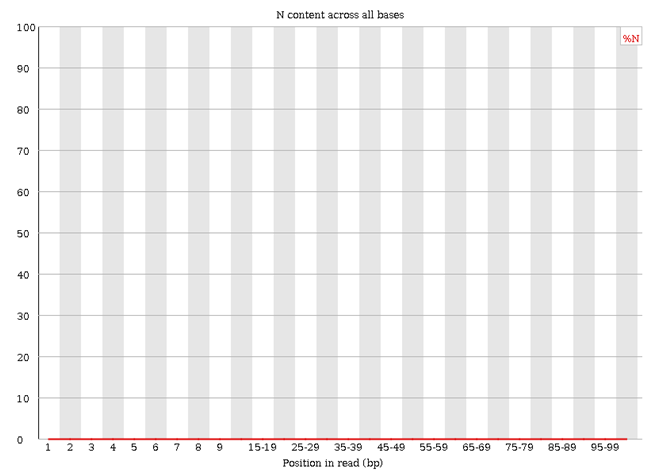

Supplement: Figure S1 — Data quality control using FastQC. (DOCX) [file pone.0066267.s001.docx]
