# Supplementary material for: Comparative Transcriptome Analysis of Adipose Tissues Reveals that ECM-Receptor Interaction Is Involved in the Depot-Specific Adipogenesis in Cattle
Source: PLoS One. 2013 Jun 21;8(6):e66267. doi: 10.1371/journal.pone.0066267 (PMC3689780; doi:10.1371/journal.pone.0066267)
Supplement: Table S1 — The primer sequence of DEG used for qRT-PCR analysis. (DOCX) [file pone.0066267.s002.docx]

Table S1. **The primer sequence of DEG used for qRT-PCR analysis.**

| **Gene** | **Accession No.** | **Primer sequence (5` to 3`)** | | **Annealing  Temperature** | **Product size** |
| --- | --- | --- | --- | --- | --- |
| ***TNMD*** | ENSBTAG00000021059 | F | CTCTTATTGTCCTGTTTTGGGGG | 56 °C | 105 bp |
|  |  | R | TCTTCTTCTTCTCTCCATTGCTGT |  |  |
| ***DUSP27*** | ENSBTAG00000008652 | F | GGTCAAGGAAGATGAGGATGAGG | 58 °C | 133bp |
|  |  | R | CAGGGAGGAGAAGGTGTCTGTTT |  |  |
| ***CCL2*** | ENSBTAG00000037811 | F | CCCTCCTGTGCCTGCTACTC | 66 °C | 158 bp |
|  |  | R | CTTGCTGCTGGTGACTCTTCTGT |  |  |
| ***PPP1R3C*** | ENSBTAG00000014831 | F | GGGGACTGTGAAGGTGAAGAACA | 58 °C | 159 bp |
|  |  | R | GGTAGGTCAATGGCAAACGAGAA |  |  |
| ***TPM2*** | ENSBTAG00000011424 | F | AGAGTTTGCTGAAAGGTCGGTG | 58 °C | 187 bp |
|  |  | R | ATGGAAAGGAGAAGAGAGAGGGG |  |  |
| ***CD163*** | ENSBTAG00000019669 | F | AGTCCCATCTTTCACTCTGCCC | 66 °C | 145 bp |
|  |  | R | TTGACCTCCACTCTTCCTTCACA |  |  |
| ***ITGAM*** | ENSBTAG00000047238 | F | ATCCAATCTACTCCAGCAACCCA | 58 °C | 141 bp |
|  |  | R | GAGACAAACCTCTTCATCCGCTC |  |  |
| ***CCR2*** | ENSBTAG00000013586 | F | AAGGGGAATGATGAAGAACCCA | 58 °C | 138 bp |
|  |  | R | GCCCACAAAACCAAAGATGAAGA |  |  |
| ***FASN*** | ENSBTAG00000015980 | F | GTGCGTCCTGGTGTCTAACCTC | 58 °C | 169 bp |
|  |  | R | CTCCTCGGGCTTGTCTTGTTC |  |  |
| ***DGAT2*** | ENSBTAG00000001154 | F | GCCAAGGAAAGACACAGAGGAAA | 58 °C | 173 bp |
|  |  | R | AGCACAGGAACACTGCCACC |  |  |
| ***UCP1*** | ENSBTAG00000004647 | F | CCTAATGACTGGAGGCGTGG | 58 °C | 98 bp |
|  |  | R | GAGGTTTGGGACCGTGGAGA |  |  |
| ***ISL1*** | ENSBTAG00000001241 | F | ACCTGCTATGCCGCTAACCC | 58 °C | 134 bp |
|  |  | R | ATGATGCTCCGCTTCTTATCCTT |  |  |
| ***TCF21*** | ENSBTAG00000021827 | F | AACCCGAGAGTGACCTGAAAGAA | 58 °C | 164 bp |
|  |  | R | AGGGGGAAGCAGAGACAGAGAG |  |  |
| ***MMP9*** | ENSBTAG00000020676 | F | TGAGGGTAAGGTGCTGCTGTTC | 60 °C | 141 bp |
|  |  | R | TACTGAAAGATGTCGTGCGTGCT |  |  |
| ***BDNF*** | ENSBTAG00000008134 | F | CCCCCTCTCCTCTTCCTGCT | 58 °C | 135bp |
|  |  | R | CACCCACTCGCTGATGCTGT |  |  |
| ***TBX15*** | ENSBTAG00000007767 | F | CTGCTCCTTTGACACTCACGCT | 58 °C | 171 bp |
|  |  | R | AACATCCTCCTGCCTGCTTTG |  |  |
| ***SLPI*** | ENSBTAG00000004148 | F | GTGGTGTGGGAGTTCCATT | 58 °C | 106 bp |
|  |  | R | CAGACATTTGTTGGCTTCTTCTG |  |  |
| ***ZIC1*** | ENSBTAG00000014751 | F | AAAAGGACGCACACAGGGGA | 64 °C | 126 bp |
|  |  | R | GCAAAGATAGGGCTTGTCGCT |  |  |
| ***MFSD2A*** | ENSBTAG00000013054 | F | CGGCGGCAGAACAAGAAGG | 58 °C | 183 bp |
|  |  | R | GGAGCAGGCAAGCGGG |  |  |
| ***FGF7*** | ENSBTAG00000004013 | F | TCAAAAGGGGGTTCCAGTAAGAG | 58 °C | 157 bp |
|  |  | R | GAAAAGATGAAAAAGAAAACAGTCCA |  |  |
| ***FASN*** | ENSBTAG00000015980 | F | GTGCGTCCTGGTGTCTAACCTC | 58 °C | 169 bp |
|  |  | R | CTCCTCGGGCTTGTCTTGTTC |  |  |
| ***TBX5*** | ENSBTAG00000011384 | F | GCACCAAGAGGAAAGATGAAGAA | 58 °C | 106 bp |
|  |  | R | TAGCCAGAACGGTAGAAGGGGT |  |  |
| ***CYP17A1*** | ENSBTAG00000014335 | F | GCCCCATCTATTCCTTTCGTTT | 58 °C | 156 bp |
|  |  | R | CAATGCCCTTTTGGTTGTCTG |  |  |
| ***LHX8*** | ENSBTAG00000019192 | F | TGCTCAGGACAACAACCCAGA | 64 °C | 147 bp |
|  |  | R | GGGGCAGAGGAGGAGTGATTAG |  |  |
| ***EN1*** | ENSBTAG00000021494 | F | ACTGACTCGCAGCAACCCCT | 58 °C | 124 bp |
|  |  | R | GCTTGTCCTCCTTCTCGTTCTTC |  |  |
| ***EEF1A2*** | ENSBTAG00000021685 | F | CTCGGCTCTGGACTCACTGCT | 64 °C | 101 bp |
|  |  | R | TCTCCTTGCCCATTCTGCCT |  |  |
| ***ADAM23*** | ENSBTAG00000019694 | F | AAAGGCAAGACACCAGCAAAAAC | 58 °C | 134 bp |
|  |  | R | CCACATAATCAGAAGACAGCAAACC |  |  |
| ***ERBB3*** | ENSBTAG00000010444 | F | GCTTTGCCTGCCGACTCTT | 60 °C | 139 bp |
|  |  | R | CTGGCTACACACACTCCTCCATAC |  |  |
| ***IGF2BP2*** | ENSBTAG00000007666 | F | CTGTGCCAATGCCGAAATAGAG | 60 °C | 202 bp |
|  |  | R | AGCGAAAGGGTGGTAGGGGG |  |  |
| ***STRA6*** | ENSBTAG00000007348 | F | CAGCCTCTACCACACCTGCCT | 64 °C | 147 bp |
|  |  | R | CTGTCCCCAGTCAAGAAATCCAC |  |  |
| ***GAPDH*** | ENSBTAG00000014731 | F | CAAGATGGTGAAGGTCGGAGTG | 58 °C | 158 bp |
|  |  | R | CGTGGGTGGAATCATACTGGAA |  |  |
| ***ACTB*** | ENSBTAG00000026199 | F | GGACCTCTACGCCAACACGG | 58 °C | 160 bp |
|  |  | R | GGAGCCGCCAATCCACAC |  |  |
