# Supplementary material for: Comparative Transcriptome Analysis of Adipose Tissues Reveals that ECM-Receptor Interaction Is Involved in the Depot-Specific Adipogenesis in Cattle
Source: PLoS One. 2013 Jun 21;8(6):e66267. doi: 10.1371/journal.pone.0066267 (PMC3689780; doi:10.1371/journal.pone.0066267)
Supplement: Table S2 — RNA-seq reads and mapping rate of different adipose depots from nine Hanwoo individuals. (DOCX) [file pone.0066267.s003.docx]

**Table S2. RNA-seq reads and mapping rate of different adipose depots from nine Hanwoo individuals.**

|  |  | Adipose tissue | | | | | |
| --- | --- | --- | --- | --- | --- | --- | --- |
| Animal ID | No. of | Subcutaneous | | intramuscular | | Omental | |
| 1 | total reads | 34,961,934 |  | 32,795,970 |  | 40,145,136 |  |
|  | reads after QC | 34,956,668 |  | 32,791,028 |  | 40,139,167 |  |
|  | accepted_Hit | 33,887,004 | (96.94%) | 32,079,358 | (97.83%) | 39,471,224 | (98.34%) |
| 2 | total reads | 37,902,974 |  | 38,957,018 |  | 41,075,552 |  |
|  | reads after QC | 37,865,228 |  | 38,917,997 |  | 41,034,551 |  |
|  | accepted_Hit | 36,992,627 | (97.70%) | 38,194,374 | (98.14%) | 40,914,740 | (99.71%) |
| 3 | total reads | 31,844,692 |  | 27,859,998 |  | 36,559,594 |  |
|  | reads after QC | 31,835,650 |  | 27,852,084 |  | 36,549,104 |  |
|  | accepted_Hit | 30,785,146 | (96.70%) | 26,789,222 | (96.18%) | 35,924,487 | (98.29%) |
| 4 | total reads | 29,063,304 |  | 38,983,416 |  | 49,285,572 |  |
|  | reads after QC | 29,058,910 |  | 38,977,599 |  | 49,278,117 |  |
|  | accepted_Hit | 28,119,540 | (96.77%) | 38,573,994 | (98.96%) | 47,590,598 | (96.58%) |
| 5 | total reads | 36,503,342 |  | 42,173,502 |  | 37,910,536 |  |
|  | reads after QC | 36,492,884 |  | 42,161,496 |  | 37,899,902 |  |
|  | accepted_Hit | 35,224,619 | (96.52%) | 41,159,915 | (97.62%) | 36,510,193 | (96.33%) |
| 6 | total reads | 37,683,440 |  | 36,733,112 |  | 33,236,374 |  |
|  | reads after QC | 37,645,749 |  | 36,696,313 |  | 33,203,354 |  |
|  | accepted_Hit | 36,578,016 | (97.16%) | 36,183,925 | (98.60%) | 32,295,059 | (97.26%) |
| 7 | total reads | 40,603,752 |  | 45,547,786 |  | 33,840,910 |  |
|  | reads after QC | 40,597,681 |  | 45,540,853 |  | 33,835,854 |  |
|  | accepted_Hit | 39,131,638 | (96.39%) | 45,472,436 | (99.85%) | 32,628,247 | (96.43%) |
| 8 | total reads | 31,265,648 |  | 25,404,790 |  | 33,774,810 |  |
|  | reads after QC | 31,234,834 |  | 25,379,357 |  | 33,741,132 |  |
|  | accepted_Hit | 30,588,262 | (97.93%) | 25,094,287 | (98.88%) | 32,878,392 | (97.44%) |
| 9 | total reads | 36,409,346 |  | 33,896,998 |  | 37,094,812 |  |
|  | reads after QC | 36,398,844 |  | 33,887,341 |  | 37,083,976 |  |
|  | accepted_Hit | 35,037,306 | (96.26%) | 33,013,485 | (97.42%) | 35,835,326 | (96.63%) |
| Average | total reads | 35,137,604 |  | 35,816,954 |  | 38,102,588 |  |
|  | reads after QC | 35,120,716 |  | 35,800,452 |  | 38,085,017 |  |
|  | accepted_Hit | 34,038,240 | (96.92%) | 35,173,444 | (98.25%) | 37,116,474 | (97.46%) |
