# Supplementary material for: Comparative Transcriptome Analysis of Adipose Tissues Reveals that ECM-Receptor Interaction Is Involved in the Depot-Specific Adipogenesis in Cattle
Source: PLoS One. 2013 Jun 21;8(6):e66267. doi: 10.1371/journal.pone.0066267 (PMC3689780; doi:10.1371/journal.pone.0066267)
Supplement: Table S3 — Quantitative RT-PCR validation of DEGs in each adipose tissue. (DOCX) [file pone.0066267.s004.docx]

**Table S3. Quantitative RT-PCR validation of DEGs in each adipose tissue.**

1. Comparison of the relative expression level of DEGs in intramuscular fat compared to other tissues between RNA-seq and quantitative RT-PCR

|  | RNA-seq result^*^ | |  | Realtime PCR (ACTB)^**^ | |  | Realtime PCR (GAPDH)^**^ | |
| --- | --- | --- | --- | --- | --- | --- | --- | --- |
|  | Omental fat^†^ | Subcutaneous fat^†^ |  | Omental fat^†^ | Subcutaneous fat^†^ |  | Omental fat^†^ | Subcutaneous fat^†^ |
| Up-regulated gene |  |  |  |  |  |  |  |  |
| *TNMD* | 7.582795541 | 7.290738932 |  | 6.761740741 | 7.295366667 |  | 6.641433333 | 7.544677778 |
| *DUSP23* | 8.637712761 | 6.723681197 |  | 1.978833333 | 2.262546296 |  | 1.858525926 | 2.511857407 |
| *CCL2* | 3.648650536 | 3.331055979 |  | 3.538559259 | 3.3062 |  | 3.418251852 | 3.555511111 |
| *PPP1R3C* | 2.622462215 | 2.348394392 |  | 1.917725926 | 1.371337037 |  | 1.797418519 | 1.620648148 |
| *TPM2* | 3.255892524 | 3.458496831 |  | 7.958037037 | 6.863340741 |  | 7.83772963 | 7.112651852 |
| Down-regulated gene |  |  |  |  |  |  |  |  |
| *CD163* | -2.59290445 | -2.370162009 |  | -3.8217 | -4.162562963 |  | -3.942007407 | -3.913251852 |
| *ITGAM* | -3.178046367 | -2.068014834 |  | -2.769344444 | -3.382377778 |  | -2.889651852 | -3.133066667 |
| *CCR2* | -2.943209311 | -2.549572772 |  | -2.63277037 | -2.1849 |  | -2.753077778 | -1.935588889 |
| *FASN* | -2.070953687 | -2.71168049 |  | -2.486314815 | -3.4197 |  | -2.606622222 | -3.170388889 |
| *DGAT* | -2.188408802 | -2.519565518 |  | -3.194677778 | -3.961274074 |  | -3.314985185 | -3.711962963 |

| ^*^Results are presented by log_2_(fold-chage) |
| --- |
| ^**^Results are presented by log_2_(fold-chage) and ACTB and GAPDH were used as a reference gene for relative expression level analysis (Livak and Schmittgen, 2001) |
| †Each tissue indicates pair-wisely compared tissue with intramuscular fat and the values represent the relative mRNA abundance of intramuscular fat to other tissues indicated |
|  |
| Reference |
| Livak, K.J., and Schmittgen, T.D. (2001) Analysis of relative gene expression data using real-time quantitative PCR and the 2(-Delta Delta C(T)) Method. *Methods* **25**(4), 402-8 |

1. Comparison of the relative expression level of DEGs in Omental fat compared to other tissues between RNA-seq and quantitative RT-PCR

|  | RNA-seq result^*^ | |  | Realtime PCR (ACTB)^**^ | |  | Realtime PCR (GAPDH)^**^ | |
| --- | --- | --- | --- | --- | --- | --- | --- | --- |
|  | Subcutaneous fat^†^ | Intramuscular fat^†^ |  | Subcutaneous fat^†^ | Intramuscular fat^†^ |  | Subcutaneous fat^†^ | Intramuscular fat† |
| Up-regulated gene |  |  |  |  |  |  |  |  |
| *UCP1* | 2.257996125 | 3.160584312 |  | 2.177892593 | 3.060014815 |  | 2.547511111 | 3.180322222 |
| *ISL1* | 5.216295643 | 3.692037537 |  | 3.262251852 | 5.431211111 |  | 3.63187037 | 5.551518519 |
| *TCF21* | 4.292913048 | 8.191062054 |  | 4.975537037 | 5.525188889 |  | 5.345155556 | 5.645496296 |
| *MMP9* | 3.721998041 | 3.07950315 |  | 4.432566667 | 5.672437037 |  | 4.802185185 | 5.792744444 |
| *BDNF* | 4.906165587 | 2.304811471 |  | 2.91722963 | 5.106418519 |  | 3.286848148 | 5.226725926 |
| Down-regulated gene |  |  |  |  |  |  |  |  |
| *TBX15* | -7.491262798 | -7.5859685 |  | -6.425588889 | -6.175848148 |  | -6.05597037 | -6.055540741 |
| *SLP1* | -3.214617058 | -3.277696653 |  | -0.987540741 | -0.634966667 |  | -0.617922222 | -0.514659259 |
| *ZIC1* | -8.733341983 | -6.710274081 |  | -8.793475926 | -11.48877222 |  | -8.423857407 | -11.36846481 |
| *MFSD2A* | -3.289699568 | -2.234958978 |  | -1.227333333 | -3.157837037 |  | -0.857714815 | -3.03752963 |
| *FGF7* | -3.036326305 | -2.24726629 |  | -1.186385185 | -1.646651852 |  | -0.816766667 | -1.526344444 |

| ^*^Results are presented by log_2_(fold-chage) |
| --- |
| ^**^Results are presented by log_2_(fold-chage) and ACTB and GAPDH were used as a reference gene for relative expression level analysis (Livak and Schmittgen, 2001) |
| †Each tissue indicates pair-wisely compared tissue with omental fat and the values represent the relative mRNA abundance of omental fat to other tissues indicated |
|  |
| Reference |
| Livak, K.J., and Schmittgen, T.D. (2001) Analysis of relative gene expression data using real-time quantitative PCR and the 2(-Delta Delta C(T)) Method. *Methods* **25**(4), 402-8 |

1. Comparison of the relative expression level of DEGs in Omental fat compared to other tissues between RNA-seq and quantitative RT-PCR

|  | RNA-seq result^*^ | |  | Realtime PCR (ACTB)^**^ | |  | Realtime PCR (GAPDH)^**^ | |
| --- | --- | --- | --- | --- | --- | --- | --- | --- |
|  | Omental fat^†^ | Intramuscular fat^†^ |  | Omental fat^†^ | Intramuscular fat^†^ |  | Omental fat^†^ | Intramuscular fat^†^ |
| Up-regulated gene |  |  |  |  |  |  |  |  |
| *FASN* | 0.672233058 | 2.71168049 |  | 0.933385185 | 3.4197 |  | 0.563766667 | 3.170388889 |
| *TBX5* | 5.201957617 | 5.037736051 |  | 6.10717963 | 6.60372037 |  | 5.737561111 | 6.354409259 |
| *CYP17A2* | 4.159184867 | 3.462433762 |  | 4.781205556 | 5.0999 |  | 4.411587037 | 4.850588889 |
| *LHX8* | 7.898169436 | 2.617482926 |  | 6.498331481 | 2.382275926 |  | 6.128712963 | 2.132964815 |
| *EN1* | 3.044432763 | 2.67248675 |  | 2.982903704 | 4.068583333 |  | 2.613285185 | 3.819272222 |
| Down-regulated gene |  |  |  |  |  |  |  |  |
| *EEF1A2* | -1.96014702 | -6.768295424 |  | -3.3046 | -8.901351852 |  | -3.674218519 | -9.150662963 |
| *ADAM23* | -3.783880587 | -3.346282232 |  | -0.462996296 | -0.369144444 |  | -0.832614815 | -0.618455556 |
| *ERBB2* | -3.347889708 | -2.826071438 |  | -3.763451852 | -2.683862963 |  | -4.13307037 | -2.933174074 |
| *IGF2BP* | -3.326063567 | -4.609580722 |  | -3.950553704 | -4.910912963 |  | -4.320172222 | -5.160224074 |
| *STRA6* | -3.260136201 | -3.134006554 |  | -4.667518519 | -4.430244444 |  | -5.037137037 | -4.679555556 |

| ^*^Results are presented by log_2_(fold-chage) |
| --- |
| ^**^Results are presented by log_2_(fold-chage) and ACTB and GAPDH were used as a reference gene for relative expression level analysis (Livak and Schmittgen, 2001) |
| †Each tissue indicates pair-wisely compared tissue with subcutaneous fat and the values represent the relative mRNA abundance of subcutaneous fat to other tissues indicated |
|  |
| Reference |
| Livak, K.J., and Schmittgen, T.D. (2001) Analysis of relative gene expression data using real-time quantitative PCR and the 2(-Delta Delta C(T)) Method. *Methods* **25**(4), 402-8 |
