# Supplementary material for: Comparative Transcriptome Analysis of Adipose Tissues Reveals that ECM-Receptor Interaction Is Involved in the Depot-Specific Adipogenesis in Cattle
Source: PLoS One. 2013 Jun 21;8(6):e66267. doi: 10.1371/journal.pone.0066267 (PMC3689780; doi:10.1371/journal.pone.0066267)
Supplement: Table S4 — Statistical analysis of the correlation between RNAseq and qRT-PCR. (DOCX) [file pone.0066267.s005.docx]

**Table S4. Statistical analysis of the correlation between RNAseq and qRT-PCR.**

|  | Correlation | P-value |
| --- | --- | --- |
| Intramuscular | 0.8547347 | 1.63E-03 |
| omental | 0.9636408 | 7.32E-06 |
| subcutaneous | 0.9519825 | 2.19E-05 |
