# Supplementary material for: Comparative Transcriptome Analysis of Adipose Tissues Reveals that ECM-Receptor Interaction Is Involved in the Depot-Specific Adipogenesis in Cattle
Source: PLoS One. 2013 Jun 21;8(6):e66267. doi: 10.1371/journal.pone.0066267 (PMC3689780; doi:10.1371/journal.pone.0066267)
Supplement: Table S5 — Biological process GO terms of DEGs resulted from pairwise comparison among three different adipose depots. (DOCX) [file pone.0066267.s006.docx]

**Table S5. Biological process GO terms of DEGs resulted from pairwise comparison among three different adipose depots.**

| **Categories** | **GO ID** | **Terms** | **Count** | **P-value** |
| --- | --- | --- | --- | --- |
| OS |  |  |  |  |
| Subcutaneous | GO:0032502 | developmental process | 213 | 6.23E-12 |
|  | GO:0032501 | multicellular organismal process | 258 | 2.92E-09 |
|  | GO:0050896 | response to stimulus | 212 | 1.70E-07 |
|  | GO:0022610 | biological adhesion | 57 | 1.25E-05 |
|  | GO:0040011 | locomotion | 39 | 4.10E-05 |
|  | GO:0002376 | immune system process | 65 | 1.65E-03 |
| Omental | GO:0032502 | developmental process | 326 | 9.74E-16 |
|  | GO:0022610 | biological adhesion | 102 | 1.33E-12 |
|  | GO:0032501 | multicellular organismal process | 398 | 4.04E-12 |
|  | GO:0002376 | immune system process | 120 | 3.16E-09 |
|  | GO:0050896 | response to stimulus | 310 | 9.24E-07 |
|  | GO:0040011 | locomotion | 55 | 1.96E-05 |
|  | GO:0051179 | localization | 254 | 3.04E-04 |
|  | GO:0016043 | cellular component organization | 213 | 8.88E-04 |
|  | GO:0051234 | establishment of localization | 219 | 4.07E-03 |
|  | GO:0016265 | death | 70 | 4.97E-03 |
| OI |  |  |  |  |
| Intramuscular | GO:0032502 | developmental process | 753 | 1.51E-47 |
|  | GO:0032501 | multicellular organismal process | 881 | 5.97E-29 |
|  | GO:0065007 | biological regulation | 1324 | 3.10E-16 |
|  | GO:0022610 | biological adhesion | 189 | 9.16E-16 |
|  | GO:0016265 | death | 160 | 9.10E-07 |
|  | GO:0040011 | locomotion | 98 | 4.68E-05 |
|  | GO:0009987 | cellular process | 1686 | 2.34E-04 |
|  | GO:0040007 | growth | 46 | 7.21E-04 |
|  | GO:0016043 | cellular component organization | 437 | 9.01E-04 |
|  | GO:0044085 | cellular component biogenesis | 182 | 8.79E-03 |
| Omental | GO:0002376 | immune system process | 224 | 6.06E-15 |
|  | GO:0008152 | metabolic process | 1094 | 9.56E-03 |
|  | GO:0050896 | response to stimulus | 521 | 9.99E-03 |
| IS |  |  |  |  |
| Subcutaneous | GO:0002376 | immune system process | 211 | 2.70E-16 |
|  | GO:0008152 | metabolic process | 1021 | 1.49E-05 |
|  | GO:0050896 | response to stimulus | 484 | 1.00E-03 |
| Intramuscular | GO:0032502 | developmental process | 755 | 2.30E-49 |
|  | GO:0032501 | multicellular organismal process | 879 | 1.54E-29 |
|  | GO:0065007 | biological regulation | 1332 | 1.79E-19 |
|  | GO:0022610 | biological adhesion | 187 | 2.14E-15 |
|  | GO:0016265 | death | 163 | 1.39E-07 |
|  | GO:0009987 | cellular process | 1698 | 1.18E-06 |
|  | GO:0040011 | locomotion | 104 | 1.29E-06 |
|  | GO:0016043 | cellular component organization | 447 | 5.74E-05 |
|  | GO:0048511 | rhythmic process | 34 | 1.37E-03 |
|  | GO:0040007 | growth | 43 | 3.80E-03 |
| IC |  |  |  |  |
| Intramuscular | GO:0032502 | developmental process | 727 | 1.28E-42 |
|  | GO:0032501 | multicellular organismal process | 850 | 5.84E-25 |
|  | GO:0065007 | biological regulation | 1306 | 1.79E-17 |
|  | GO:0022610 | biological adhesion | 180 | 1.10E-13 |
|  | GO:0016265 | death | 158 | 7.71E-07 |
|  | GO:0040011 | locomotion | 103 | 1.34E-06 |
|  | GO:0009987 | cellular process | 1672 | 5.94E-06 |
|  | GO:0016043 | cellular component organization | 441 | 7.53E-05 |
|  | GO:0040007 | growth | 42 | 5.27E-03 |
| Combined O & S | GO:0002376 | immune system process | 234 | 2.33E-18 |
|  | GO:0008152 | metabolic process | 1121 | 7.36E-05 |
|  | GO:0050896 | response to stimulus | 530 | 1.99E-03 |
|  | GO:0009987 | cellular process | 1481 | 7.99E-03 |
|  | GO:0051234 | establishment of localization | 402 | 8.58E-03 |
