# Supplementary material for: Comparative Transcriptome Analysis of Adipose Tissues Reveals that ECM-Receptor Interaction Is Involved in the Depot-Specific Adipogenesis in Cattle
Source: PLoS One. 2013 Jun 21;8(6):e66267. doi: 10.1371/journal.pone.0066267 (PMC3689780; doi:10.1371/journal.pone.0066267)
Supplement: Table S6 — GO terms of cellular components and molecular function of depot specific DEGs. (DOCX) [file pone.0066267.s007.docx]

**Table S6. GO terms of cellular components and molecular function of depot specific DEGs**

1. **Up-regulated DEGs**

|  | **GO ID** | **Term** | **PValue** |
| --- | --- | --- | --- |
| **Omental fat** |  |  |  |
| Cellualr component | GO:0005576 | extracellular region | 7.59E-03 |
|  | GO:0044421 | extracellular region part | 1.55E-02 |
|  | GO:0044464 | cell part | 2.13E-02 |
|  | GO:0005623 | cell | 2.16E-02 |
| Molecular Function | GO:0060089 | molecular transducer activity | 8.44E-04 |
| **Intramuscular Fat** |  |  |  |
| Cellualr component | GO:0044421 | extracellular region part | 8.01E-10 |
|  | GO:0045202 | synapse | 1.41E-06 |
|  | GO:0044456 | synapse part | 2.35E-05 |
|  | GO:0005576 | extracellular region | 3.95E-03 |
| Molecular Function | GO:0005488 | binding | 3.03E-18 |
|  | GO:0005198 | structural molecule activity | 1.33E-08 |
|  | GO:0030528 | transcription regulator activity | 1.51E-07 |
|  | GO:0030234 | enzyme regulator activity | 2.62E-03 |
| **Subcutaneous fat** |  |  |  |
| Cellular component | GO:0005576 | extracellular region | 4.03E-07 |
|  | GO:0044421 | extracellular region part | 6.10E-07 |
| Molecular Function | GO:0003824 | catalytic activity | 2.25E-03 |
|  | GO:0009055 | electron carrier activity | 3.11E-03 |
|  | GO:0016209 | antioxidant activity | 3.63E-03 |
|  | GO:0005215 | transporter activity | 2.69E-02 |

1. **Down-regulated DEGs**

|  | **GO ID** | **Term** | **PValue** |
| --- | --- | --- | --- |
| **Omental fat** |  |  |  |
| Cellular component | GO:0044421 | extracellular region part | 1.02E-16 |
|  | GO:0005576 | extracellular region | 1.01E-14 |
| Molecular Function | GO:0005488 | binding | 1.47E-02 |
|  | GO:0005198 | structural molecule activity | 1.93E-02 |
|  | GO:0060089 | molecular transducer activity | 4.57E-02 |
| **Intramuscular fat** |  |  |  |
| Cellular component | GO:0044464 | cell part | 8.45E-06 |
|  | GO:0005623 | cell | 8.77E-06 |
|  | GO:0031975 | envelope | 1.37E-04 |
| Molecular Function | GO:0003824 | catalytic activity | 5.64E-16 |
|  | GO:0009055 | electron carrier activity | 1.08E-04 |
|  | GO:0005488 | binding | 4.26E-02 |
| **Subcutaneous fat** |  |  |  |
| Cellular component | GO:0005576 | extracellular region | 2.10E-13 |
|  | GO:0044421 | extracellular region part | 4.72E-12 |
|  | GO:0045202 | synapse | 1.87E-05 |
|  | GO:0044456 | synapse part | 1.37E-03 |
| Molecular Function | GO:0005198 | structural molecule activity | 3.12E-05 |
|  | GO:0060089 | molecular transducer activity | 2.53E-04 |
|  | GO:0005488 | binding | 2.61E-04 |
