# Supplementary material for: Comparative Transcriptome Analysis of Adipose Tissues Reveals that ECM-Receptor Interaction Is Involved in the Depot-Specific Adipogenesis in Cattle
Source: PLoS One. 2013 Jun 21;8(6):e66267. doi: 10.1371/journal.pone.0066267 (PMC3689780; doi:10.1371/journal.pone.0066267)
Supplement: Table S7 — KEGG pathways of DEGs resulted from the pairwise comparison among three different adipose depots. (DOCX) [file pone.0066267.s008.docx]

**Table S7. KEGG pathways of DEGs resulted from pairwise comparison among three different adipose depots.**

1. **KEGG pathways of genes up-regulated in omental fat compared to intramuscular fat**

| **KEGG_A_level** | **KEGG_B_level** | **Term** | **Count** | **PValue** |
| --- | --- | --- | --- | --- |
| Cellular Processes | Transport and Catabolism | Lysosome | 45 | 2.59E-09 |
| Environmental Information Processing | Membrane Transport | ABC transporters | 14 | 1.27E-02 |
| Human Diseases | Immune Diseases | Primary immunodeficiency | 13 | 4.48E-03 |
| Metabolism | Amino Acid Metabolism | Histidine metabolism | 10 | 2.60E-02 |
|  | Amino Acid Metabolism | Lysine degradation | 13 | 3.03E-02 |
|  | Amino Acid Metabolism | Tryptophan metabolism | 12 | 3.52E-02 |
|  | Amino Acid Metabolism | Valine, leucine and isoleucine degradation | 27 | 3.39E-11 |
|  | Carbohydrate Metabolism | Butanoate metabolism | 15 | 2.60E-04 |
|  | Carbohydrate Metabolism | Citrate cycle (TCA cycle) | 11 | 1.46E-02 |
|  | Carbohydrate Metabolism | Propanoate metabolism | 17 | 4.63E-06 |
|  | Carbohydrate Metabolism | Pyruvate metabolism | 15 | 1.76E-03 |
|  | Energy Metabolism | Sulfur metabolism | 6 | 2.66E-02 |
|  | Lipid Metabolism | Biosynthesis of unsaturated fatty acids | 10 | 3.52E-03 |
|  | Lipid Metabolism | Fatty acid elongation in mitochondria | 7 | 2.79E-04 |
|  | Lipid Metabolism | Fatty acid metabolism | 23 | 7.38E-09 |
|  | Lipid Metabolism | Glycerolipid metabolism | 14 | 1.54E-02 |
|  | Lipid Metabolism | Steroid hormone biosynthesis | 14 | 1.85E-02 |
|  | Metabolism of Cofactors and Vitamins | Pantothenate and CoA biosynthesis | 7 | 1.90E-02 |
|  | Metabolism of Other Amino Acids | beta-Alanine metabolism | 8 | 4.17E-02 |
|  | Xenobiotics Biodegradation and Metabolism | Metabolism of xenobiotics by cytochrome P450 | 17 | 1.68E-02 |
| Organismal Systems | Endocrine System | PPAR signaling pathway | 33 | 8.91E-10 |
|  | Immune System | B cell receptor signaling pathway | 20 | 1.69E-02 |
|  | Immune System | Complement and coagulation cascades | 23 | 4.38E-04 |
|  | Immune System | Fc gamma R-mediated phagocytosis | 26 | 3.95E-03 |
|  | Immune System | Hematopoietic cell lineage | 22 | 1.87E-02 |
|  | Immune System | T cell receptor signaling pathway | 25 | 3.70E-02 |

1. **KEGG pathways of genes up-regulated in the subcutaneous fat compared to intramuscular fat**

| **KEGG_A_level** | **KEGG_B_level** | **Term** | **Count** | **PValue** |
| --- | --- | --- | --- | --- |
| Cellular Processes | Transport and Catabolism | Lysosome | 38 | 9.27E-07 |
| Environmental Information Processing | Signaling Molecules and Interaction | Cell adhesion molecules (CAMs) | 31 | 5.13E-03 |
| Human Diseases | Immune Diseases | Primary immunodeficiency | 12 | 7.18E-03 |
| Metabolism | Amino Acid Metabolism | Histidine metabolism | 11 | 4.92E-03 |
|  | Amino Acid Metabolism | Lysine degradation | 13 | 1.69E-02 |
|  | Amino Acid Metabolism | Tryptophan metabolism | 14 | 2.57E-03 |
|  | Amino Acid Metabolism | Valine, leucine and isoleucine degradation | 30 | 4.49E-15 |
|  | Carbohydrate Metabolism | Butanoate metabolism | 14 | 4.50E-04 |
|  | Carbohydrate Metabolism | Citrate cycle (TCA cycle) | 12 | 2.49E-03 |
|  | Carbohydrate Metabolism | Propanoate metabolism | 18 | 2.31E-07 |
|  | Carbohydrate Metabolism | Pyruvate metabolism | 13 | 7.65E-03 |
|  | Glycan Biosynthesis and Metabolism | Glycosaminoglycan degradation | 8 | 2.17E-02 |
|  | Glycan Biosynthesis and Metabolism | Other glycan degradation | 8 | 4.05E-03 |
|  | Lipid Metabolism | Biosynthesis of unsaturated fatty acids | 11 | 4.06E-04 |
|  | Lipid Metabolism | Fatty acid elongation in mitochondria | 7 | 1.76E-04 |
|  | Lipid Metabolism | Fatty acid metabolism | 18 | 1.18E-05 |
|  | Lipid Metabolism | Glycerolipid metabolism | 14 | 7.92E-03 |
|  | Lipid Metabolism | Steroid hormone biosynthesis | 14 | 9.65E-03 |
|  | Metabolism of Other Amino Acids | Glutathione metabolism | 16 | 3.01E-03 |
|  | Metabolism of Terpenoids and Polyketides | Limonene and pinene degradation | 7 | 8.78E-03 |
|  | Xenobiotics Biodegradation and Metabolism | Drug metabolism | 16 | 2.42E-02 |
|  | Xenobiotics Biodegradation and Metabolism | Metabolism of xenobiotics by cytochrome P450 | 18 | 3.20E-03 |
| Organismal Systems | Endocrine System | PPAR signaling pathway | 21 | 1.06E-03 |
|  | Immune System | B cell receptor signaling pathway | 22 | 1.30E-03 |
|  | Immune System | Complement and coagulation cascades | 21 | 1.06E-03 |
|  | Immune System | Fc epsilon RI signaling pathway | 20 | 1.13E-02 |
|  | Immune System | Fc gamma R-mediated phagocytosis | 23 | 1.23E-02 |
|  | Immune System | Hematopoietic cell lineage | 25 | 6.37E-04 |
|  | Immune System | Natural killer cell mediated cytotoxicity | 28 | 3.23E-02 |
|  | Immune System | T cell receptor signaling pathway | 24 | 2.78E-02 |

1. **KEGG pathways of genes up-regulated in intramuscular fat compared to omental fat**

| **KEGG_A_level** | **KEGG_B_level** | **Term** | **Count** | **PValue** |
| --- | --- | --- | --- | --- |
| Cellular Processes | Cell Communication | Focal adhesion | 71 | 2.22E-10 |
|  | Cell Communication | Tight junction | 34 | 1.28E-02 |
|  | Cell Growth and Death | Apoptosis | 24 | 1.52E-02 |
|  | Cell Growth and Death | p53 signaling pathway | 20 | 1.46E-02 |
|  | Cell Motility | Regulation of actin cytoskeleton | 60 | 4.66E-05 |
| Environmental Information Processing | Signal Transduction | Calcium signaling pathway | 57 | 5.15E-07 |
|  | Signal Transduction | Hedgehog signaling pathway | 17 | 1.94E-02 |
|  | Signal Transduction | MAPK signaling pathway | 71 | 4.74E-05 |
|  | Signal Transduction | Phosphatidylinositol signaling system | 20 | 3.45E-02 |
|  | Signal Transduction | TGF-beta signaling pathway | 27 | 1.70E-03 |
|  | Signal Transduction | Wnt signaling pathway | 36 | 2.62E-02 |
|  | Signaling Molecules and Interaction | Cell adhesion molecules (CAMs) | 38 | 7.82E-04 |
|  | Signaling Molecules and Interaction | Cytokine-cytokine receptor interaction | 58 | 2.06E-02 |
|  | Signaling Molecules and Interaction | ECM-receptor interaction | 37 | 1.43E-08 |
| Genetic Information Processing | Translation | Ribosome | 54 | 1.15E-20 |
| Human Diseases | Cancers | Bladder cancer | 14 | 1.73E-02 |
|  | Cancers | Chronic myeloid leukemia | 21 | 2.07E-02 |
|  | Cancers | Pathways in cancer | 89 | 1.69E-06 |
|  | Cancers | Small cell lung cancer | 28 | 3.97E-04 |
|  | Cardiovascular Diseases | Arrhythmogenic right ventricular cardiomyopathy (ARVC) | 36 | 2.22E-09 |
|  | Cardiovascular Diseases | Dilated cardiomyopathy | 48 | 2.73E-14 |
|  | Cardiovascular Diseases | Hypertrophic cardiomyopathy (HCM) | 47 | 2.94E-15 |
|  | Cardiovascular Diseases | Viral myocarditis | 20 | 2.29E-02 |
|  | Endocrine and Metabolic Diseases | Type II diabetes mellitus | 18 | 1.08E-03 |
| Organismal Systems | Circulatory System | Cardiac muscle contraction | 25 | 1.63E-03 |
|  | Circulatory System | Vascular smooth muscle contraction | 40 | 2.49E-06 |
|  | Development | Axon guidance | 39 | 2.25E-04 |
|  | Endocrine System | Adipocytokine signaling pathway | 18 | 4.87E-02 |
|  | Endocrine System | Insulin signaling pathway | 33 | 2.42E-02 |
|  | Environmental Adaptation | Circadian rhythm | 8 | 2.53E-03 |
|  | Excretory System | Aldosterone-regulated sodium reabsorption | 13 | 3.35E-02 |
|  | Immune System | Leukocyte transendothelial migration | 29 | 3.30E-02 |
|  | Immune System | NOD-like receptor signaling pathway | 22 | 7.99E-04 |
|  | Nervous System | Long-term potentiation | 19 | 2.93E-02 |

1. **KEGG pathways of genes up-regulated in intramuscular fat compared to subcutaneous fat**

| **KEGG_A_level** | **KEGG_B_level** | **Term** | **Count** | **PValue** |
| --- | --- | --- | --- | --- |
| Cellular Processes | Cell Communication | Focal adhesion | 68 | 1.39E-08 |
|  | Cell Communication | Tight junction | 38 | 1.74E-03 |
|  | Cell Growth and Death | Apoptosis | 27 | 2.51E-03 |
|  | Cell Growth and Death | p53 signaling pathway | 20 | 1.90E-02 |
|  | Cell Motility | Regulation of actin cytoskeleton | 59 | 1.94E-04 |
| Environmental Information Processing | Signal Transduction | Calcium signaling pathway | 58 | 5.23E-07 |
|  | Signal Transduction | Hedgehog signaling pathway | 16 | 4.93E-02 |
|  | Signal Transduction | MAPK signaling pathway | 82 | 5.24E-08 |
|  | Signal Transduction | Phosphatidylinositol signaling system | 21 | 2.35E-02 |
|  | Signal Transduction | TGF-beta signaling pathway | 30 | 1.93E-04 |
|  | Signal Transduction | Wnt signaling pathway | 41 | 2.68E-03 |
|  | Signaling Molecules and Interaction | Cell adhesion molecules (CAMs) | 32 | 4.09E-02 |
|  | Signaling Molecules and Interaction | Cytokine-cytokine receptor interaction | 64 | 2.75E-03 |
|  | Signaling Molecules and Interaction | ECM-receptor interaction | 32 | 1.21E-05 |
| Genetic Information Processing | Translation | Ribosome | 44 | 3.94E-12 |
| Human Diseases | Cancers | Basal cell carcinoma | 17 | 2.07E-02 |
|  | Cancers | Chronic myeloid leukemia | 21 | 2.70E-02 |
|  | Cancers | Pathways in cancer | 90 | 2.82E-06 |
|  | Cancers | Small cell lung cancer | 25 | 6.75E-03 |
|  | Cardiovascular Diseases | Arrhythmogenic right ventricular cardiomyopathy (ARVC) | 39 | 4.65E-11 |
|  | Cardiovascular Diseases | Dilated cardiomyopathy | 47 | 3.99E-13 |
|  | Cardiovascular Diseases | Hypertrophic cardiomyopathy (HCM) | 46 | 4.89E-14 |
|  | Cardiovascular Diseases | Viral myocarditis | 20 | 2.95E-02 |
|  | Endocrine and Metabolic Diseases | Type II diabetes mellitus | 17 | 4.06E-03 |
| Organismal Systems | Circulatory System | Cardiac muscle contraction | 28 | 1.57E-04 |
|  | Circulatory System | Vascular smooth muscle contraction | 42 | 6.19E-07 |
|  | Development | Axon guidance | 43 | 1.46E-05 |
|  | Endocrine System | Melanogenesis | 26 | 2.81E-02 |
|  | Environmental Adaptation | Circadian rhythm | 7 | 1.51E-02 |
|  | Excretory System | Aldosterone-regulated sodium reabsorption | 14 | 1.74E-02 |
|  | Immune System | Chemokine signaling pathway | 43 | 3.91E-02 |
|  | Immune System | NOD-like receptor signaling pathway | 18 | 3.07E-02 |
|  | Nervous System | Long-term potentiation | 20 | 1.90E-02 |

**(e) KEGG pathways of genes up-regulated in omental fat compared to subcutaneous fat**

| **KEGG_A_level** | **KEGG_B_level** | **Term** | **Count** | **PValue** |
| --- | --- | --- | --- | --- |
| Cellular Processes | Transport and Catabolism | Lysosome | 20 | 2.59E-03 |
| Environmental Information Processing | Signal Transduction | Calcium signaling pathway | 23 | 2.73E-02 |
|  | Signaling Molecules and Interaction | Cytokine-cytokine receptor interaction | 34 | 7.01E-03 |
|  | Signaling Molecules and Interaction | Neuroactive ligand-receptor interaction | 32 | 1.52E-02 |
| Human Diseases | Cardiovascular Diseases | Dilated cardiomyopathy | 14 | 3.42E-02 |
| Metabolism | Lipid Metabolism | Fatty acid metabolism | 12 | 2.41E-04 |
| Organismal Systems | Circulatory System | Vascular smooth muscle contraction | 24 | 2.44E-05 |
|  | Development | Axon guidance | 22 | 1.52E-03 |
|  | Endocrine System | Adipocytokine signaling pathway | 11 | 4.29E-02 |
|  | Endocrine System | PPAR signaling pathway | 21 | 2.84E-07 |
|  | Immune System | Chemokine signaling pathway | 24 | 2.88E-02 |

**(f) KEGG pathways of genes up-regulated in subcutaneous fat compared to omental fat**

| **KEGG_A_level** | **KEGG_B_level** | **Term** | **Count** | **PValue** |
| --- | --- | --- | --- | --- |
| Cellular Processes | Cell Communication | Focal adhesion | 23 | 3.18E-04 |
| Environmental Information Processing | Membrane Transport | ABC transporters | 8 | 5.24E-03 |
|  | Signaling Molecules and Interaction | Cell adhesion molecules (CAMs) | 17 | 6.98E-04 |
|  | Signaling Molecules and Interaction | Cytokine-cytokine receptor interaction | 22 | 1.81E-02 |
|  | Signaling Molecules and Interaction | ECM-receptor interaction | 18 | 4.65E-07 |
| Human Diseases | Cardiovascular Diseases | Viral myocarditis | 9 | 2.27E-02 |
|  | Immune Diseases | Asthma | 6 | 1.26E-02 |
|  | Immune Diseases | Graft-versus-host disease | 6 | 4.11E-02 |
| Organismal Systems | Circulatory System | Vascular smooth muscle contraction | 11 | 4.87E-02 |
|  | Development | Axon guidance | 13 | 2.46E-02 |
|  | Endocrine System | Renin-angiotensin system | 5 | 8.29E-03 |
|  | Immune System | Complement and coagulation cascades | 11 | 1.85E-03 |

**(g) KEGG pathways of genes up-regulated in the instramuscular fat compared to the combined omental and subcutaneous fat**

| **KEGG_A_level** | **KEGG_B_level** | **Term** | **Count** | **PValue** |
| --- | --- | --- | --- | --- |
| Cellular Processes | Cell communication | Focal adhesion | 70 | 7.26E-10 |
|  | Cell communication | Tight junction | 36 | 4.21E-03 |
|  | Cell growth and death | Apoptosis | 26 | 3.90E-03 |
|  | Cell growth and death | p53 signaling pathway | 19 | 3.03E-02 |
|  | Cell motility | Regulation of actin cytoskeleton | 62 | 1.27E-05 |
| Environmental Information Processing | Signal transduction | Calcium signaling pathway | 52 | 3.56E-05 |
|  | Signal transduction | MAPK signaling pathway | 77 | 9.04E-07 |
|  | Signal transduction | Phosphatidylinositol signaling system | 21 | 1.86E-02 |
|  | Signal transduction | TGF-beta signaling pathway | 31 | 4.76E-05 |
|  | Signal transduction | Wnt signaling pathway | 39 | 5.78E-03 |
|  | Signaling molecules and interaction | Cell adhesion molecules (CAMs) | 31 | 4.95E-02 |
|  | Signaling molecules and interaction | Cytokine-cytokine receptor interaction | 62 | 4.06E-03 |
|  | Signaling molecules and interaction | ECM-receptor interaction | 35 | 2.14E-07 |
| Genetic Information Processing | Translation | Ribosome | 51 | 5.72E-18 |
| Human Diseases | Cancers | Bladder cancer | 13 | 4.09E-02 |
|  | Cancers | Chronic myeloid leukemia | 21 | 2.15E-02 |
|  | Cancers | Pathways in cancer | 88 | 3.78E-06 |
|  | Cancers | Small cell lung cancer | 28 | 4.20E-04 |
|  | Cardiovascular diseases | Arrhythmogenic right ventricular cardiomyopathy (ARVC) | 37 | 5.42E-10 |
|  | Cardiovascular diseases | Dilated cardiomyopathy | 48 | 3.14E-14 |
|  | Cardiovascular diseases | Hypertrophic cardiomyopathy (HCM) | 48 | 5.73E-16 |
|  | Cardiovascular diseases | Viral myocarditis | 21 | 1.17E-02 |
|  | Endocrine and metabolic diseases | Type II diabetes mellitus | 17 | 3.20E-03 |
| Organismal Systems | Circulatory system | Cardiac muscle contraction | 27 | 2.81E-04 |
|  | Circulatory system | Vascular smooth muscle contraction | 41 | 9.61E-07 |
|  | Development | Axon guidance | 43 | 8.07E-06 |
|  | Endocrine system | Insulin signaling pathway | 32 | 4.10E-02 |
|  | Environmental adaptation | Circadian rhythm | 7 | 1.35E-02 |
|  | Excretory system | Aldosterone-regulated sodium reabsorption | 14 | 1.45E-02 |
|  | Immune system | Chemokine signaling pathway | 44 | 1.80E-02 |
|  | Immune system | NOD-like receptor signaling pathway | 21 | 2.17E-03 |
|  | Nervous system | Long-term potentiation | 19 | 3.03E-02 |
|  | Nervous system | Neurotrophin signaling pathway | 30 | 3.78E-02 |

**(h) KEGG pathways of genes up-regulated in the combined omental and subcutaneous fat compared to the intramuscular fat**

| **KEGG_A_level** | **KEGG_B_level** | **Term** | **Count** | **PValue** |
| --- | --- | --- | --- | --- |
| Cellular Processes | Transport and catabolism | Lysosome | 47 | 3.46E-10 |
| Environmental Information Processing | Membrane transport | ABC transporters | 13 | 3.40E-02 |
| Human Diseases | Immune diseases | Primary immunodeficiency | 12 | 1.48E-02 |
| Metabolism | Amino acid metabolism | Histidine metabolism | 12 | 2.99E-03 |
| Metabolism | Amino acid metabolism | Lysine degradation | 17 | 6.25E-04 |
| Metabolism | Amino acid metabolism | Tryptophan metabolism | 14 | 6.11E-03 |
| Metabolism | Amino acid metabolism | Valine, leucine and isoleucine degradation | 30 | 6.27E-14 |
| Metabolism | Carbohydrate metabolism | Butanoate metabolism | 15 | 3.10E-04 |
| Metabolism | Carbohydrate metabolism | Citrate cycle (TCA cycle) | 13 | 1.59E-03 |
| Metabolism | Carbohydrate metabolism | Propanoate metabolism | 18 | 9.71E-07 |
| Metabolism | Carbohydrate metabolism | Pyruvate metabolism | 15 | 2.07E-03 |
| Metabolism | Energy metabolism | Sulfur metabolism | 6 | 2.84E-02 |
| Metabolism | Glycan biosynthesis and metabolism | Glycosaminoglycan degradation | 8 | 3.54E-02 |
| Metabolism | Glycan biosynthesis and metabolism | Other glycan degradation | 7 | 2.85E-02 |
| Metabolism | Lipid metabolism | Biosynthesis of unsaturated fatty acids | 11 | 9.03E-04 |
| Metabolism | Lipid metabolism | Fatty acid elongation in mitochondria | 7 | 3.07E-04 |
| Metabolism | Lipid metabolism | Fatty acid metabolism | 21 | 3.86E-07 |
| Metabolism | Lipid metabolism | Glycerolipid metabolism | 15 | 6.96E-03 |
| Metabolism | Lipid metabolism | Steroid hormone biosynthesis | 15 | 8.60E-03 |
| Metabolism | Metabolism of cofactors and vitamins | Pantothenate and CoA biosynthesis | 7 | 2.05E-02 |
| Metabolism | Metabolism of other amino acids | beta-Alanine metabolism | 8 | 4.51E-02 |
| Metabolism | Metabolism of other amino acids | Glutathione metabolism | 15 | 1.84E-02 |
| Metabolism | Metabolism of terpenoids and polyketides | Limonene and pinene degradation | 7 | 1.41E-02 |
| Metabolism | Xenobiotics biodegradation and metabolism | Metabolism of xenobiotics by cytochrome P450 | 19 | 3.66E-03 |
| Organismal Systems | Endocrine system | PPAR signaling pathway | 27 | 6.49E-06 |
| Organismal Systems | Immune system | B cell receptor signaling pathway | 21 | 9.65E-03 |
| Organismal Systems | Immune system | Complement and coagulation cascades | 23 | 5.53E-04 |
| Organismal Systems | Immune system | Fc epsilon RI signaling pathway | 20 | 2.94E-02 |
| Organismal Systems | Immune system | Fc gamma R-mediated phagocytosis | 26 | 4.92E-03 |
| Organismal Systems | Immune system | Hematopoietic cell lineage | 22 | 2.22E-02 |
| Organismal Systems | Immune system | T cell receptor signaling pathway | 26 | 2.55E-02 |
