# Supplementary material for: Comparative Transcriptome Analysis of Adipose Tissues Reveals that ECM-Receptor Interaction Is Involved in the Depot-Specific Adipogenesis in Cattle
Source: PLoS One. 2013 Jun 21;8(6):e66267. doi: 10.1371/journal.pone.0066267 (PMC3689780; doi:10.1371/journal.pone.0066267)
Supplement: Table S8 — Summary of the enriched KEGG pathway results based on the depot specific DEGs. (DOCX) [file pone.0066267.s009.docx]

**Table S8. Summary of enriched KEGG pathway results based on the depot specific DEGs.**

1. **KEGG pathway of DEGs up-regulated in each depot**

| **Tissue** | **KEGG ID** | **KEGG Term** | **P-Value** | **Genes** |
| --- | --- | --- | --- | --- |
| Omental fat | hsa04142 | Lysosome | 1.67E-05 | *TCIRG1, CTSZ, LIPA, GM2A, ACP5, CTSA, PPT1, ACP2, DNASE2, SLC11A1, CD68, LAPTM5, TPP1, CTSB, CTSH, ATP6V0D2* |
|  | hsa00071 | Fatty acid metabolism | 4.90E-04 | *ACADVL, CPT1B, ACSL1, ACADM, EHHADH, ADH6, ACADL, HADHB* |
|  | hsa03320 | PPAR signaling pathway | 7.59E-04 | *LPL, CPT1B, ACSL1, ACADM, OLR1, EHHADH, UCP1, ACADL, DBI, PCK1* |
|  | hsa04670 | Leukocyte transendothelial migration | 1.05E-03 | *ITGAL, NCF2, NCF1, CLDN3, NCF4, MMP9, ITGB2, ITGA4, ITGAM, CLDN15, CYBB, RAC2, CLDN1* |
| Intramuscular | hsa05410 | Hypertrophic cardiomyopathy (HCM) | 6.66E-16 | *TNF, MYL2, MYL3, TNNC1, MYBPC3, ITGB4, ITGA11, TGFB3, CACNB1, TPM2, TTN, ITGB1, TPM4, ACTG1, ACE, DES, ITGB8, ITGAV, DMD, CACNA2D1, ACTC1, IL6, SLC8A1, CACNG7, CACNG6, ITGA1, LMNA, ITGA2, MYH7, ITGA3, MYH6, CACNG1, CACNA1S, ITGA9, ATP2A2, SGCG, ITGA5, ITGA8, ITGA7, SGCD, RYR2, CACNA1C* |
|  | hsa05414 | Dilated cardiomyopathy | 3.40E-15 | *ADCY2, TNF, MYL2, MYL3, ADCY8, TNNC1, MYBPC3, ITGB4, ITGA11, TGFB3, CACNB1, TPM2, TTN, ITGB1, TPM4, ACTG1, DES, ITGB8, ITGAV, DMD, CACNA2D1, ACTC1, SLC8A1, CACNG7, CACNG6, ITGA1, LMNA, ITGA2, MYH7, ITGA3, MYH6, CACNG1, CACNA1S, ITGA9, ATP2A2, SGCG, ITGA5, ITGA8, PLN, ITGA7, SGCD, RYR2, CACNA1C* |
|  | hsa04510 | Focal adhesion | 1.07E-10 | *PGF, PDGFA, BCAR1, VCL, CHAD, ACTG1, PAK4, ZYX, COL11A2, COL11A1, AKT3, SHC4, BRAF, ACTN4, MYLK2, ACTN1, PRKCG, ACTN2, PPP1CC, FLNC, FLNA, SPDYA, VEGFC, CCND1, CCND2, JUN, VEGFA, RELN, LAMC2, CAV3, MYL2, TNC, ITGB4, ITGA11, COL2A1, ITGB1, IGF1R, COL6A6, ITGB8, ITGAV, COMP, PPP1R12A, PIK3R3, THBS1, THBS4, FLT1, FLT4, ITGA1, ITGA2, MYLPF, ITGA3, BIRC3, BIRC2, COL4A6, LAMA1, ITGA9, ITGA5, ITGA8, ITGA7, MYLK* |
|  | hsa05412 | Arrhythmogenic right ventricular cardiomyopathy (ARVC) | 2.58E-09 | *ITGB4, ITGA11, CACNB1, ITGB1, ACTG1, DES, ITGB8, DMD, ITGAV, SLC8A1, CACNA2D1, ACTN4, CACNG7, CACNG6, ITGA1, LMNA, ITGA2, ACTN1, ITGA3, ACTN2, CACNG1, CACNA1S, ITGA9, ATP2A2, SGCG, ITGA5, ITGA8, ITGA7, SGCD, RYR2, CACNA1C* |
|  | hsa04020 | Calcium signaling pathway | 3.02E-08 | *ADCY2, TNNC2, TNNC1, ADCY8, ITPKB, ATP2B3, ATP2B4, PLCB4, GRIN2C, NOS3, PTGER1, HTR4, MYLK2, PRKCG, PLCE1, ADRB2, GNAQ, CHRM3, RYR3, RYR1, RYR2, ADORA2B, ERBB4, CYSLTR2, ADORA2A, PHKA1, BDKRB1, BDKRB2, HRH1, PDE1B, HRH2, PLCD4, PPP3CC, CAMK2B, TRPC1, SLC8A1, SLC8A2, ITPR3, PTGFR, CACNA1S, ITPR1, ATP2A2, PLN, ATP2A1, AVPR1A, CACNA1H, CACNA1C, HTR2B, MYLK, HTR2A* |
|  | hsa04270 | Vascular smooth muscle contraction | 4.18E-08 | *MYL6, KCNMB4, ADCY2, ADORA2B, ADORA2A, ADCY8, CALD1, PPP1R12B, MRVI1, PRKG1, KCNMB1, ACTG2, PLCB4, MYL6B, PPP1R12A, CALCRL, RAMP1, PPP1R14A, KCNMA1, RAMP3, RAMP2, BRAF, ACTA2, MYLK2, PRKCG, ITPR3, PRKCE, PPP1CC, CACNA1S, ITPR1, SPDYA, PLA2G4A, GNAQ, MYH11, AVPR1A, CACNA1C, MYLK* |
|  | hsa04010 | MAPK signaling pathway | 6.71E-07 | *FGF18, FGF7, PDGFA, FGF16, TGFB3, NFKB1, NFKB2, FOS, CASP3, MAP3K8, IL1B, FAS, MYC, MAP2K6, AKT3, IL1A, BRAF, CACNG7, RELB, CACNG6, PRKCG, CACNG1, MECOM, FLNC, FLNA, DDIT3, RASGRF2, JUN, GADD45G, GADD45B, GADD45A, IL1R1, FGFR4, TNF, FGFR3, CACNB1, MAPKAPK2, SRF, ATF2, ELK4, MAP3K2, DUSP14, DUSP16, HSPA6, PPP3CC, NFATC2, HSPA8, RASA2, CACNA2D1, NTF3, MAP2K3, NR4A1, CACNA1S, DUSP5, PLA2G4A, DUSP3, RPS6KA3, MAPK12, DUSP1, CACNA1H, MAPK8IP1, CACNA1C, DUSP8* |
|  | hsa05200 | Pathways in cancer | 2.64E-05 | *FGF18, FGF7, PTGS2, PDGFA, PGF, FGF16, TGFB3, NFKB1, NFKB2, GLI2, GLI1, FOS, CASP3, SLC2A1, RALA, HHIP, RARB, FAS, WNT6, MYC, AKT3, RET, PLD1, HSP90AA1, BRAF, RUNX1T1, PRKCG, CDK6, MECOM, DAPK3, VEGFC, CCND1, HIF1A, JUN, VEGFA, WNT9B, LAMC2, WNT11, BID, FGFR3, EGLN3, KITLG, NFKBIA, ITGB1, IGF1R, ITGAV, AXIN2, RUNX1, PIK3R3, TRAF5, IL6, IL8, SMAD3, ITGA2, ITGA3, BIRC3, BIRC2, COL4A6, STAT3, FZD7, RALGDS, WNT2B, LAMA1, CDKN1A, CBLB, CDKN1B, ETS1, RASSF1* |
|  | hsa04810 | Regulation of actin cytoskeleton | 1.68E-04 | *FGF18, FGFR4, FGF7, FGFR3, MYL2, PDGFA, WASF1, BCAR1, SSH2, FGF16, ITGB4, ITGA11, PIP5K1B, ABI2, BDKRB1, PIP5K1A, BDKRB2, ITGB1, VCL, ACTG1, TIAM2, ITGB8, ITGAV, PAK4, PPP1R12A, PIK3R3, ARHGEF4, BRAF, ACTN4, ARHGEF7, ITGA1, ITGA2, ACTN1, MYLK2, MYLPF, IGF2, ACTN2, ITGA3, PPP1CC, SPDYA, ITGA9, CHRM3, ITGA5, ITGA8, ITGA7, F2, MYLK* |
|  | hsa04930 | Type II diabetes mellitus | 1.44E-03 | *PRKCZ, IRS2, TNF, SOCS2, SOCS3, SOCS1, HK1, SOCS4, IGF2, PRKCE, KCNJ11, MAFA, PIK3R3, CACNA1C, ABCC8* |
|  | hsa04360 | Axon guidance | 5.02E-03 | *ABLIM2, EFNA1, PLXNA2, L1CAM, EPHB3, ITGB1, EPHB1, SEMA5A, CXCR4, SEMA3G, PAK4, SEMA7A, NFAT5, SEMA3D, PPP3CC, RHOD, NFATC2, NFATC1, NGEF, EFNB2, DPYSL5, EPHA2, EPHA3, SEMA6A, RND1, RGS3, SEMA4C, SEMA4A* |
|  | hsa04350 | TGF-beta signaling pathway | 5.05E-03 | *NOG, SMAD9, TNF, LTBP1, SMAD7, FST, BMPR2, TGFB3, SMAD3, INHBA, ID2, PPP2CA, COMP, SMURF2, BMP7, THBS1, BMPR1B, MYC, BMP6, THBS4, PITX2* |
|  | hsa05222 | Small cell lung cancer | 7.44E-03 | *PTGS2, NFKBIA, ITGA2, CDK6, ITGA3, NFKB1, BIRC3, ITGB1, BIRC2, COL4A6, LAMA1, CCND1, CDKN1B, ITGAV, LAMC2, RARB, PIK3R3, TRAF5, MYC, AKT3* |
|  | hsa04530 | Tight junction | 8.52E-03 | *PRKCZ, CLDN19, MYH15, MYL2, CASK, CLDN11, ACTG1, PPP2CA, PPP2R2B, AKT3, ACTN4, MYH1, MPDZ, MYH2, MYLPF, ACTN1, PRKCG, ACTN2, MYH7, MYH6, CLDN20, PRKCE, CSDA, MYH11, PARD6G, JAM3, MYH7B, PPP2R2A* |
|  | hsa04720 | Long-term potentiation | 9.16E-03 | *BRAF, ADCY8, PRKCG, PPP1CC, ITPR3, ITPR1, SPDYA, RPS6KA3, PLCB4, GNAQ, GRIN2C, GRIA1, PPP1R1A, PPP1R12A, PPP3CC, CAMK2B, CACNA1C* |
|  | hsa04070 | Phosphatidylinositol signaling system | 9.42E-03 | *PIP5K1B, DGKH, PRKCG, ITPKB, PIP5K1A, ITPR3, ITPR1, PLCE1, PLCB4, DGKB, INPP5J, DGKD, DGKG, PLCD4, SYNJ2, INPP4B, PIK3R3, INPP5A* |
| Subcutaneous | hsa04610 | Complement and coagulation cascades | 4.59E-03 | *MASP1, F13A1, C1S, C2, PROS1, C8G* |
|  | hsa00480 | Glutathione metabolism | 7.89E-03 | *MGST3, GSS, GSTT1, ANPEP, MGST1* |

1. **KEGG pathway of DEGs down-regulated in each depot**

| **Tissue** | **KEGG ID** | **KEGG Term** | **P-Value** | **Gene** |
| --- | --- | --- | --- | --- |
| Omental fat | hsa04512 | ECM-receptor interaction | 3.60E-05 | *LAMA2, COL6A6, COL6A3, COL1A2, ITGB4, ITGA11, SV2B, THBS3, THBS4, FN1* |
|  | hsa05200 | Pathways in cancer | 1.86E-03 | *WNT10B, BMP2, IL6, FGF7, FGF11, PRKCG, MMP2, LAMA2, WNT2, FOS, CBLB, RASSF5, JUN, SLC2A1, ABL1, FN1* |
|  | hsa04510 | Focal adhesion | 1.96E-03 | *LAMA2, COL6A6, PAK3, JUN, COL6A3, COL1A2, ITGB4, ITGA11, PRKCG, THBS3, THBS4, FN1* |
|  | hsa04514 | Cell adhesion molecules (CAMs) | 4.44E-03 | *CADM3, MPZ, PVRL3, CLDN5, CD276, CLDN10, VCAN, CLDN11, SELE* |
| Intramuscular | hsa00280 | Valine, leucine and isoleucine degradation | 4.97E-12 | *BCKDHA, ACAA2, ALDH6A1, HSD17B10, ACADSB, BCAT2, ACADS, EHHADH, IL4I1, ECHS1, ACAT1, HIBADH, HADHA, HADHB, MCCC2, MUT, OXCT1, MCCC1, DLD, HADH, PCCB, PCCA, HMGCL* |
|  | hsa04142 | Lysosome | 2.31E-06 | *SGSH, AP1B1, ACP5, CTSA, PPT1, ASAH1, GLB1, SLC11A1, CD68, NAGPA, AP1S1, LAPTM5, GNPTAB, MAN2B1, IDUA, GBA, CTSZ, PLA2G15, LIPA, GUSB, NAPSA, CTSS, GNS, LAMP3, IGF2R, GAA, CTSC, AP4B1, CTSH* |
|  | hsa00640 | Propanoate metabolism | 1.66E-05 | *ALDH6A1, SUCLG1, EHHADH, ACACA, ECHS1, ACSS2, ACSS3, ACAT1, HADHA, MUT, MLYCD, PCCB, PCCA* |
|  | hsa03320 | PPAR signaling pathway | 1.57E-04 | *ME1, ACOX2, LPL, CPT2, RXRA, EHHADH, PPARG, RXRG, AQP7, PCK2, ADIPOQ, ACOX3, ACSL1, CYP27A1, PLIN1, FABP4, PLTP, ACSL5* |
|  | hsa00071 | Fatty acid metabolism | 2.03E-04 | *ACAA2, ACADSB, CPT2, ACADS, EHHADH, ECHS1, ACAT1, HADHA, HADHB, ACOX3, ACSL1, HADH, ACSL5* |
|  | hsa05340 | Primary immunodeficiency | 2.37E-04 | *CIITA, PTPRC, CD8A, CD3D, CD8B, CD3E, UNG, LCK, CD4, JAK3, BLNK, BTK* |
|  | hsa04660 | T cell receptor signaling pathway | 3.61E-04 | *PIK3CG, PTPRC, PTPN6, CD3G, VAV3, CD3D, CD8A, CD8B, CD3E, PIK3CD, MALT1, VAV1, PAK6, CARD11, LAT, RASGRP1, LCK, PIK3R5, CD4, GRAP2, PIK3R1, AKT2, LCP2* |
|  | hsa04662 | B cell receptor signaling pathway | 4.59E-04 | *PIK3CG, PTPN6, VAV3, CR2, PIK3CD, MALT1, VAV1, BTK, CARD11, RAC2, CD22, PIK3R5, PIK3AP1, INPP5D, PIK3R1, AKT2, SYK, BLNK* |
|  | hsa00650 | Butanoate metabolism | 8.20E-04 | *ACSM1, L2HGDH, ACADS, OXCT1, EHHADH, ECHS1, PDHA1, HADH, ACAT1, HMGCL, HADHA* |
|  | hsa04640 | Hematopoietic cell lineage | 2.32E-03 | *CD3G, CR2, CD3D, CD8A, CD8B, FLT3, CD3E, ANPEP, ITGA4, CD1E, ITGAM, CD2, CD22, CSF3R, EPOR, CD4, CD14, CSF1R* |
| subcutaneous | hsa04360 | Axon guidance | 3.61E-05 | *MET, L1CAM, EPHB3, NTN1, SLIT2, EPHB2, EPHA5, EPHA7, RND1, SEMA7A, SEMA3F, SEMA3D, ROBO2, EFNA4, SEMA4A* |
|  | hsa04270 | Vascular smooth muscle contraction | 6.09E-04 | *GNA13, ACTG2, ADCY2, ACTA2, ADCY8, MRVI1, MYH11, PRKG1, ITPR3, CACNA1C, ITPR1, MYLK* |
|  | hsa05412 | Arrhythmogenic right ventricular cardiomyopathy (ARVC) | 2.19E-03 | *DES, DSG2, ITGA8, DMD, DSP, RYR2, ACTN1, CACNA1C, SGCA* |
|  | hsa04916 | Melanogenesis | 3.26E-03 | *FZD8, EDNRB, WNT16, ADCY2, GNAO1, WNT5B, ADCY8, FZD2, FZD5, CAMK2A* |
|  | hsa05217 | Basal cell carcinoma | 6.54E-03 | *BMP4, FZD8, WNT16, WNT5B, FZD2, AXIN2, FZD5* |
